# Microbial consortia mediating lignocellulose turnover and denitrification in eutrophic lake sediment enrichments

**DOI:** 10.1128/msystems.00577-26

**Published:** 2026-06-29

**Authors:** Valerie C. Schiml, Kaja Stalder, Anikó Várnai, Linda L. Bergaust, Lars R. Bakken, Magnus Ø. Arntzen

**Affiliations:** 1 Faculty of Chemistry, Biotechnology and Food Science, Norwegian University of Life Sciences (NMBU), Ås, Norway; E O Lawrence Berkeley National Laboratory, Berkeley, California, USA

**Keywords:** freshwater lake, lake sediment, denitrification, lignocellulose degradation, NO dismutase

## Abstract

Lignocellulose is a major component of plant biomass and is recalcitrant, with efficient degradation typically requiring oxygen-dependent oxidative and carbohydrate-active enzymes (CAZymes). Anaerobic turnover is slower but can be supported by microbes capable of nitrate respiration, including denitrifiers and dissimilatory nitrate reduction to ammonium (DNRA) bacteria, which may use nitrate or nitric oxide as alternative oxidants. Anoxic layers beneath the oxic zones of eutrophic lake sediments, where nitrate penetrates from surface waters, provide a natural habitat for such organisms. To investigate these processes, we established nitrate-amended enrichments from organic-rich sediments of 10 eutrophic lakes and applied gas kinetics alongside metagenomics and metaproteomics to characterize the microbial communities. We identified a set of core microbial metagenome-assembled genomes (MAGs) present in all enrichments, dominated by *Pseudomonadota*, *Bacteroidota*, *Verrucomicrobiota*, and *Actinomycetota*, which played key roles in denitrification and fermentation. Lignocellulose degradation, however, was largely carried out by species outside the core microbiome—that is, different key degraders between lakes, suggesting lake-specific specialization. Among these, we observed potential respiratory DNRA pathways and a broad repertoire of CAZymes targeting various lignocellulose subfractions. Interestingly, many MAGs also encoded nitric oxide dismutases (NODs), enzymes postulated to convert NO to molecular oxygen and dinitrogen gas. Together, these findings advance our understanding of anaerobic biomass degradation and nitrogen cycling in eutrophic freshwater sediments, while highlighting the unexplored functional diversity of NOD-containing bacteria as an intriguing open question for future research.

**IMPORTANCE:**

Lignocellulose, the main structural component of plant biomass, represents a vast reservoir of organic carbon in natural environments. Although lignocellulose breakdown is commonly associated with oxygen-rich conditions, it also occurs in oxygen-depleted habitats such as lake sediments, where the responsible microbes and processes are poorly understood. This study reveals how diverse microbial communities can degrade lignocellulose while respiring nitrate, linking carbon turnover to nitrogen cycling in anoxic environments. By identifying shared and lake-specific microbial strategies, as well as a widespread but poorly characterized class of enzymes associated with nitric oxide metabolism, our work advances our understanding of anaerobic biomass degradation. These insights have implications for ecosystem functioning in nutrient-rich waters and for the development of sustainable, oxygen-free biotechnological processes.

## INTRODUCTION

Lignocellulose, a recalcitrant co-polymer of lignin, cellulose, and hemicelluloses, is typically degraded through the concerted action of specialized microorganisms that deploy diverse and often highly coordinated enzyme systems. Efficient lignocellulose depolymerization generally requires oxygen- or hydrogen peroxide-dependent oxidative enzymes—such as laccases, lignin peroxidases, and lytic polysaccharide monooxygenases (LPMOs)—working in concert with glycoside hydrolases (GHs) and other carbohydrate-active enzymes (CAZymes) (1). Because many of these key enzymes rely on molecular oxygen, lignocellulose degradation is typically far slower under anoxic conditions, where both lignin-modifying reactions (2) and LPMO-mediated cellulose decrystallization (3) are constrained by the absence of an appropriate oxidant. Nevertheless, anaerobic lignocellulose conversion remains attractive in industrial biotechnology, as it avoids costly aeration, reduces energy input, and enables integration with biogas or biofuel production, motivating the search for organisms, enzymes, and environmental conditions that can enhance this process (4).

Bacteria capable of denitrification and dissimilatory nitrate reduction to ammonium (DNRA) are compelling candidates for such anaerobic lignocellulose degradation. Denitrifiers respire nitrate (NO_3_⁻) through a stepwise reduction sequence to dinitrogen (N_2_), producing nitrite (NO_2_⁻), nitric oxide (NO), and nitrous oxide (N_2_O) as intermediates, while DNRA bacteria reduce NO_3_⁻ to ammonium (NH_4_^+^). Notably, NO is a free intermediate in denitrification and a byproduct of DNRA and could serve as an alternative electron acceptor supporting reactions that would otherwise require O_2_. Moreover, bacteria encoding nitric oxide dismutase (NOD) could, if NOD indeed catalyzes the dismutation of NO to molecular oxygen, generate molecular oxygen from NO, potentially enabling the activity of canonical lignocellulose-degrading oxidative enzymes even under bulk-anoxic conditions. These metabolic traits make denitrifiers and DNRA organisms plausible contributors to anaerobic, nitrate-linked lignocellulose depolymerization.

Environments likely to select for such organisms must be persistently anoxic yet supplied with both nitrate and lignocellulosic substrates. The oxic-anoxic interface in eutrophic lake sediments fulfills these criteria: nitrate produced or transported in the oxic surface layers can penetrate into underlying anoxic zones (5), whether by diffusion or biologically mediated transport (6), where it encounters abundant terrestrially derived organic matter. On this basis, we hypothesized that eutrophic lake sediments contain microbial populations capable of coupling lignocellulose degradation to denitrification or DNRA. To explore this possibility, we established anaerobic enrichment cultures amended with NO_3_⁻ and inoculated them with organic-rich sediments collected from several eutrophic lakes in Norway.

The enrichment cultures were monitored by meta-omics technologies, which are a powerful approach to investigate the diverse set of microorganisms within such complex communities. Metagenomics allows the detection and reassembly of microorganisms into metagenome-assembled genomes (MAGs) and the reconstruction of their potential metabolic functions individually. When combined with metaproteomics, expressed (and likely active) metabolic pathways can be highlighted for each MAG, giving insight into the ongoing functions of the microbiome, the metabolic diversity, and microbial interactions within the complex community.

Metagenomics is routinely employed to describe aquatic microbiomes (7). Within these habitats, metagenomics has made it possible to discover a new group of potential denitrifying *Planctomycetes* in deep freshwater lakes (8), as well as aligning taxonomic annotations with geochemical analysis (9) and potential carbon (C)-, nitrogen (N)-, phosphorus (P)-, and sulfur (S)-cycling pathways (10). A new level of informational depth was recently achieved by Zhang et al. in 2024, who analyzed functional genes of the N- and S-cycle in MAGs from eutrophic lake sediments, and thereby linked specific taxa with functional potential (11). Meta-omics technologies are further utilized for freshwater biomonitoring and eco-surveillance to analyze biodiversity and changes in metabolic pathways as a response to environmental cues (12, 13).

To date, mechanisms and microbial strategies for lignocellulose degradation by denitrifying microbiomes in freshwater lake sediments are scarcely represented in the literature. Song et al. linked the degradation of hemicellulose, cellulose, and lignin with various taxa in different sediment strata, and Zhang et al. isolated bacteria from a lake sediment capable of degrading submerged plant residues using endoglucanases (14, 15). To elucidate how lignocellulose is degraded in these environments, which microbial populations are responsible, and how this C-utilization is coupled to N-transformations, we established denitrifying enrichment cultures from eutrophic freshwater lake sediments. After 48 weeks of incubation, we characterized the enrichments using metagenomics and metaproteomics to assess community composition, the repertoire of carbohydrate-active enzymes (CAZymes) involved in lignocellulose turnover, and the presence and expression of enzymes supporting denitrification.

## MATERIALS AND METHODS

### Samples and enrichments

The samples studied in this work originated from freshwater lakes in Frogn and Ski municipality in Norway. Water and sediments were collected from 10 eutrophic lakes and 2 oligotrophic lakes (Table S1) using a laboratory beaker mounted on a stick. Mud from about 3–6 cm depth, lake water, and partially degraded organic matter such as leaves and wood remnants were collected and transported to the laboratory in 1-L laboratory flasks and stored at 4°C. To enrich denitrifying microorganisms capable of degrading lignocellulose under anoxic conditions, we used finely ground Norwegian spruce (*Picea abies*; roughly 40%–45% cellulose, 25%–30% hemicellulose, and 25%–28% lignin) wood as substrate for sequential batch enrichment cultures. Initial cultures were established in 120 mL serum bottles and contained 5 mL of sediment slurry together with 5 mL of lake water, 250 mg (0.5%) grinded Norwegian spruce (5 mm fraction), and were filled up to 50 mL with autoclaved H_2_O, in triplicates for all lakes. NH_4_Cl and KNO_3_ were added to give final concentrations of 2 mM and 5 mM per serum bottle, respectively. The serum bottles were capped with a butyl stopper, and the headspace was He-flushed to remove O_2_ and N_2_ (16), given a 1% O_2_ atmosphere, and incubated statically (no mixing) at 17 °C (Fig. S1A).

After 6 weeks, subcultures were made by transferring 5 mL of the initial cultures into new 120 mL serum bottles with 45 mL medium, giving a 1:10 dilution, primarily to get rid of simple carbon substrates potentially available in the sediment debris and to promote growth on complex lignocellulose. These new cultures contained fresh 0.5% Norwegian spruce, 5 mM KNO_3_, 2 mM NH_4_Cl, 5 mM KH_2_PO_4_ (pH = 6.0), and trace elements as described in previous studies (17, 18). Bottles were capped and He-washed as for the initial cultures, but without adding any O_2_. The cultures were maintained at 17°C, with no mixing for 48 weeks and with occasional monitoring of produced gases (see below and Fig. S1B). After this, the cultures were terminated, and aliquots of 7 mL were frozen at −80°C for later meta-omics analyses. It should be noted that these enrichment cultures are designed to selectively amplify microorganisms with specific metabolic capabilities and are not intended to represent the *in situ* microbial ecology of the sampled lakes. Samples were not collected at earlier time points, precluding the assessment of community compositional change during the enrichment period.

To trace microbial metabolism and denitrification activity during the enrichment period, we monitored the concentration of gases by sampling the headspaces. A temperature-controlled robotized incubation system (16) connected to a 789A GC-system (Agilent Technologies), and a chemiluminescence NO analyzer (Model 200A, Teledyne Instruments) was used to monitor and measure the headspace concentrations of NO, N_2_O, N_2_, CO_2_, and O_2_. The sampled gas was replaced by an equal volume of He to maintain the pressure. Enrichment cultures were initially provided with 5 mM KNO_3_ (=250 µmol NO_3_^−^-N bottle^−1^), and N-gas production was calculated as the sum of NO, N_2_O, and N_2_. Two more pulses of 5 mM KNO_3_ were added throughout the 48-week period (three pulses for lake 7; Table S1 and Fig. S1B). The concentrations of oxyanions (NO_3_^−^ and NO_2_^−^) in the liquid were measured by conversion to NO as described in detail by Lim et al. (19). NO_3_^−^-N mass balance, based on measured N-gases in the headspace and oxyanions in the liquid, was used to judge whether denitrification or DNRA was dominating, and the ratio between the amount of CO_2_ produced and the amount of NO_3_^-^ reduced was used to judge whether fermentative metabolism was dominating.

### Metagenomic sample preparation and data acquisition

DNA of the pellet of a 7-mL aliquot was extracted following the Quick-Start Protocol of the DNeasy PowerSoil Pro Kit (Qiagen). The pellet was dissolved in 800 µL lysis buffer from the kit, followed by a mechanical homogenization with FastPrep24 at 4.5 m/s for 45 s × 3 cycles to induce disruption of the microorganisms’ cells. The quality of DNA was analyzed with Nanodrop; DNA quantity was assessed using Qubit, and DNA degradation was carried out by electrophoresis on a 1% agarose gel. Extracted DNA was prepared and sequenced with paired ends (2 × 150 bp, 12 Gbp raw data per sample) on an Illumina NovaSeq 6000 by Novogene (UK). A negative extraction and sequencing control was included to monitor for contamination. Raw reads were trimmed, and quality control was performed with TrimGalore v0.6.6 (phred <33, length >20 bp) (https://www.bioinformatics.babraham.ac.uk/projects/trim_galore/). Co-assemblies were generated with MEGAHIT v1.2.9 (20) (*k*-mers: 21, 29, 39, 59, 79, 99, 119, and 141) from the three replicates of each lake as well as from replicates among several lakes (L1.2.6.12, L3.7, L4.5, and L8.9.10.11) based on clustering of high-quality trimmed reads with sourmash v4.8.4 (21). Individual assembly was performed on every replicate with MEGAHIT v1.2.9 (*k*-mers: 21, 29, 39, 59, 79, 99, 119, and 141) and additionally in meta-sensitive mode (*k*-mers: 21, 29, 39, 49, 59, 69, 79, 89, 99, 109, 119, 129, and 141). The assembled contigs were mapped with Minimap2 v2.17-r941 (22) and further converted to a BAM file and sorted by Samtools v1.17 (23) for metagenomic binning with MetaBAT2 v2.12.1 (24) (contigs >2,000 nt; contigs >1,500 nt from meta-sensitive individual assemblies). The same reading depth from MetaBAT2 v2.12.1 was used for binning with MaxBin2 v2.2.7 (25) (contig length >2,000 nt from co-assembly; contig length >1,000 nt from individual assembly). Dereplication for a non-redundant set of MAGs occurred in three steps with dRep v3.2.2 (26) (algorithm gANi, P_ani: 0.90, S_ani: 0.99), for which all MAGs from co- and individual assembly per lake were dereplicated, which were further combined with the co-assemblies from the clustered lakes for the second dereplication. The dereplicated set of MAGs from the second round was combined for a final dereplication, resulting in one set of MAGs for the control lakes (L4 and L5) and one set for the eutrophic lakes (L1–12 without L4 and L5). Each final set of dereplicated MAGs was taxonomically annotated with GTDB-TK v2.3.2.0 (27) and database release 214; the quality was assessed with CheckM v1.2.2 (28), and their genomes were mapped back to the metagenome high-quality trimmed reads using CoverM v0.6.1 (https://github.com/wwood/CoverM). Metabolic functions were annotated using DRAM v1.4.6 (29) with KoFam (30) database and KO list, providing identifiers from KEGG (31), UniRef90 (database v90) (32), Pfam (33), dbCan (database v11) (34), which provides CAZy IDs with description and pathway EC, and MEROPS (35), which provides peptidase IDs and scores (databases downloaded 23.01.2024). Identified CAZymes by dbCAN2 were reannotated with the dbCAN3 web server for improved CAZyme annotations. For all MAGs, module completion fractions (mcf) were quantified using the KEGG annotations and the R-package MetQy (36) to reveal their potential participation in metabolic pathways, particularly in denitrification and DNRA. The mcf was used to determine which metabolic pathway (denitrification/DNRA) was most likely used by each MAG, as indicated by a higher mcf value; this classification is a heuristic based on relative pathway completeness, not a definitive functional assignment, and individual MAG assignments carry uncertainty due to shared gene annotations, incomplete pathway recovery from assembly, and KEGG KO ambiguities—caveats that apply broadly to all genomic functional inferences in this study. The phylogenetic tree of dereplicated MAGs was created based on predicted protein-coding genes identified using Prodigal v2.6.3 (37) and analyzed using Phylophlan v.3.0.60 (38) (--min_num_markers 49). Multiple sequence alignment was performed using MAFFT v7.505 (39), and the phylogeny was inferred using RAxML v8.2.12 (40) and refined using FastTree v2.1.11 (41). The tree was visualized using iTOL v6 (42), which also enabled the integration of MAG protein abundances.

For each MAG, the average abundance per lake enrichment was calculated from the relative abundance (*n* > 0) per sample from CoverM v0.6.1. The MAG abundances were further rescaled, including the relative abundance of the unmapped reads per lake enrichment to a total of 100% to be able to compare abundances across the samples. The rescaled data were imported into R v.4.3.3, and the 200 most abundant MAGs were analyzed further with the packages tidyverse v.2.0.0 and visualized with ggplot2 v.3.4.2, while all MAGs across the lake enrichments were visualized with pheatmap v.1.0.12.

### Metaproteomic sample preparation, data acquisition, and analysis

For the extraction of proteins, frozen 7 mL sample aliquots (see above, same as those used for metagenomics) were thawed and centrifuged at 4,500 × *g* for 5 min to pellet the cells. The supernatant and the pellet were separated, and extracellular/secreted proteins in the supernatant were precipitated by adding 1 mL cold 80% trichloroacetic acid (TCA), incubated overnight at 4°C, and centrifuged at 4,500 × *g* for 30 min. The protein pellet was washed with 90% acetone in 0.01 M HCl and recentrifuged; the supernatant was discarded, and the pellet was air-dried. For the extraction of intracellular proteins, 500 µL of the lysis buffer (4% sodium dodecyl sulfate [SDS] in H_2_O) was added to the initial cell pellet and kept on ice for 30 min, followed by mechanical cell disruption by bead-beating with FastPrep24 (Thermo Fisher Scientific) and glass beads (particle size ≤106 µm; Sigma) for three cycles of 60 s at 4.5 m/s. The samples were then centrifuged for 15 min at 15,000 × *g* at 4°C, and the supernatant containing soluble intracellular proteins was used to redissolve the pellet containing extracellular proteins above. These rejoined samples were then reduced, alkylated, and digested with trypsin following the S-Trap 96-well plate digestion protocol (ProtiFi, USA) according to the manufacturer’s instructions. Peptides were eluted from the plates in 40% acetonitrile, dried in a speed vac, and redissolved in 0.1% formic acid prior to LC-MS analysis.

Peptides were analyzed by nano-LC-MS/MS using a Dionex Ultimate 3000 HPLC (Dionex, Sunnyvale, CA, USA) connected to a Q-Exactive hybrid quadrupole-orbitrap mass spectrometer (Thermo Fisher, Bremen, Germany) equipped with a nano-electrospray ion source. The peptides were loaded onto a trap column (Acclaim PepMap 100, C_18_, 5 µm, 100 Å, 300 µm i.d. × 5 mm, Thermo Scientific, Bremen, Germany) and backflushed onto an analytical column (Acclaim PepMap RCLS, C_18_, 3 µm, 100 Å, 75 µm i.d. × 50 cm, Thermo Scientific, Bremen, Germany). The flow rate was 300 nL/min, and the solvent gradient was 3%–35% B in 65 min, to 60% B in 3 min, and followed by a further increase to 80% B for column washing. Solvent A was 0.1% (vol/vol) formic acid, and solvent B was 100% (vol/vol) acetonitrile, and 0.1% (vol/vol) formic acid. The Q-Exactive mass spectrometer was operated in data-dependent mode, acquiring one full scan (400–1,500 *m*/*z*) at *R* = 70,000, followed by (up to) 12 dependent MS/MS scans at R = 35,000.

Protein identification and quantification was performed using Fragpipe v20.0 using the workflow LFQ-MBR. The predicted protein-coding genes from the metagenomic analysis were used as a reference database (5,057,886 protein sequences from all MAGs, including both eutrophic and control lake enrichments). This large database necessitated utilizing a high-end Linux computer and without software calibration of *m/z* values in the instrument RAW files; however, the mass spectrometer was externally calibrated prior to the analysis and yielded an accuracy of ~5 ppm. In addition, reversed sequences of all protein entries were concatenated to the database for estimation of false discovery rates. The tolerance level for matching to the database was 20 ppm for both MS and MS/MS. N-terminal acetylation and methionine oxidation were set as variable modifications, while carbamidomethylation of cysteines was set as fixed modifications. Trypsin was used as digestion enzyme, and one missed cleavage was allowed. Quantification was done using the LFQ Top-N (*n* = 3) algorithm, and the feature “Match between runs” was applied with an FDR of 1%. Furthermore, all final identifications were filtered to 1% FDR in Fragpipe using ProteinProphet (43), and identifications of potential contaminants and reversed sequences were removed. Proteins were considered identified if detected in at least one replicate enrichment sample for at least one lake enrichment (one non-NA value). This permissive criterion was chosen to maximize sensitivity in highly complex community samples where stochastic under sampling in data-dependent acquisition can lead to genuine proteins being detected in fewer than two of three replicates. MS/MS identification rates averaged 41% across samples (range 34%–55%) (Fig. S2), indicating that the metagenomic database adequately captured the community diversity. To calculate protein abundances per MAG, protein hits containing indistinguishable proteins originating from other species were omitted, prioritizing unique peptide evidence for MAG-level assignments consistent with best-practice guidelines and subgroup-based protein grouping recommended in CAMPI (44). LFQ values were then summed per MAG. Metagenomic annotations (taxonomy, MAG) as well as functional annotations from KoFam and dbCAN databases were propagated to the metaproteomics data.

### Detection and phylogeny of NO dismutases

Twenty previously reported NOD sequences from an alpine wetland (45) were aligned using Clustal Omega (EBI), truncated to the start of the conserved region (Y229 in DAMO_2437 in *M. oxygeniifera*), and used to generate a 339-AA-long hidden Markov model with HMMER v3.3.1. This model was then used to identify putative NODs among the predicted protein-coding genes from the metagenomic analysis (5,057,886 protein sequences) with added *M. oxygeniifera* NODs and qNOR as positive and negative controls, respectively. All potential NODs were manually inspected for an H-to-N mutation in the active site using alignments. Additionally, a few selected sequences were in-depth scrutinized for active site topology using AlphaFold3 models (46). We also analyzed our data using the HMM by Murali et al. (47) for verification of our findings; this is a 783-AA-long HMM targeting NOD. This HMM extracted the same putative NODs but with slightly different ranks and scores, underlining the specificity of our shorter HMM.

The shorter HMM was then used to identify NODs within the UniProt eubacteria sequence repository using JackHMMER with default parameters. The results were filtered to E-values < 1e^−68^ to primarily identify NODs but still allow a significant fraction of qNORs (false positives) to be present. A sequence similarity network (SSN) was constructed with the Enzyme Similarity Tool from the Enzyme Function Initiative (EFI-EST) (48) using a minimum sequence length of 600 AAs and a minimum alignment score of 95 to remove fragmented sequences. This generated multiple sequence clusters, largely distinguishing NODs from qNORs. The NOD-clusters were manually inspected for the H-to-N mutation in the active site and finally filtered so that sequences no longer present in UniProt were removed to ensure a high-quality data set. All NODs identified in the lake enrichments and NODs identified from the UniProt/SSN analysis were analyzed for phylogeny. First, redundant sequences (>95% identity) were removed, resulting in 92 unique sequences (Table S7 and S8). These were aligned with six qNOR sequences from the literature as an outgroup using MAFFT v.7.525 (EBI) (39). A phylogenetic tree was constructed using IQ-TREE v1.6.12 (49), with the best-fit substitution model selected via ModelFinder (50), and tree robustness was assessed with 1,000 ultrafast bootstrap replicates (51). The resulting tree was visualized using iTOL v7.2.2 (42) and Inkscape v1.4.2.

## RESULTS AND DISCUSSION

Eutrophic lakes contain elevated nutrient levels (N and P), and the eutrophication is further accelerated by agricultural runoff such as nitrogen-rich fertilizers. Together with falling leaves and branches in the riparian zone, this creates a niche habitat for lignocellulose-degrading denitrifying microbes. We collected samples from the shoreline sediments of 10 such eutrophic lakes and 2 control lakes (Table S1) and set up enrichment cultures on lignocellulose (ground Norwegian spruce) under denitrifying conditions with the purpose of amplifying the relative biomass of microorganisms capable of sustaining growth under such conditions and allowing for their in-depth characterization with meta-omics analysis. These enrichments showed continuous N-gas production under denitrifying conditions (Fig. S1). After 48 weeks, the cultures were terminated and analyzed. At this time, it was evident that the two control samples from lakes not exposed to nitrate leaching produced very little N-gas over the duration of the experiment (Fig. S1, Lakes 4 and 5), while the other enrichment cultures efficiently converted NO_3_^-^ to N_2_, while degrading the lignocellulose (Fig. S1, Lake 7), suggesting an increased presence of denitrifying microorganisms in the eutrophic lakes compared to the control lakes. The N mass balance calculations showed that the measured N-gas production accounted for practically all the oxyanions reduced, meaning that denitrification was the dominating respiratory pathway (Fig. S1, legend). The electron flow to denitrification largely accounted for the measured CO_2_ production in the enrichment, meaning that any fermentation products were ultimately respired (not accumulating).

**Fig 1:**
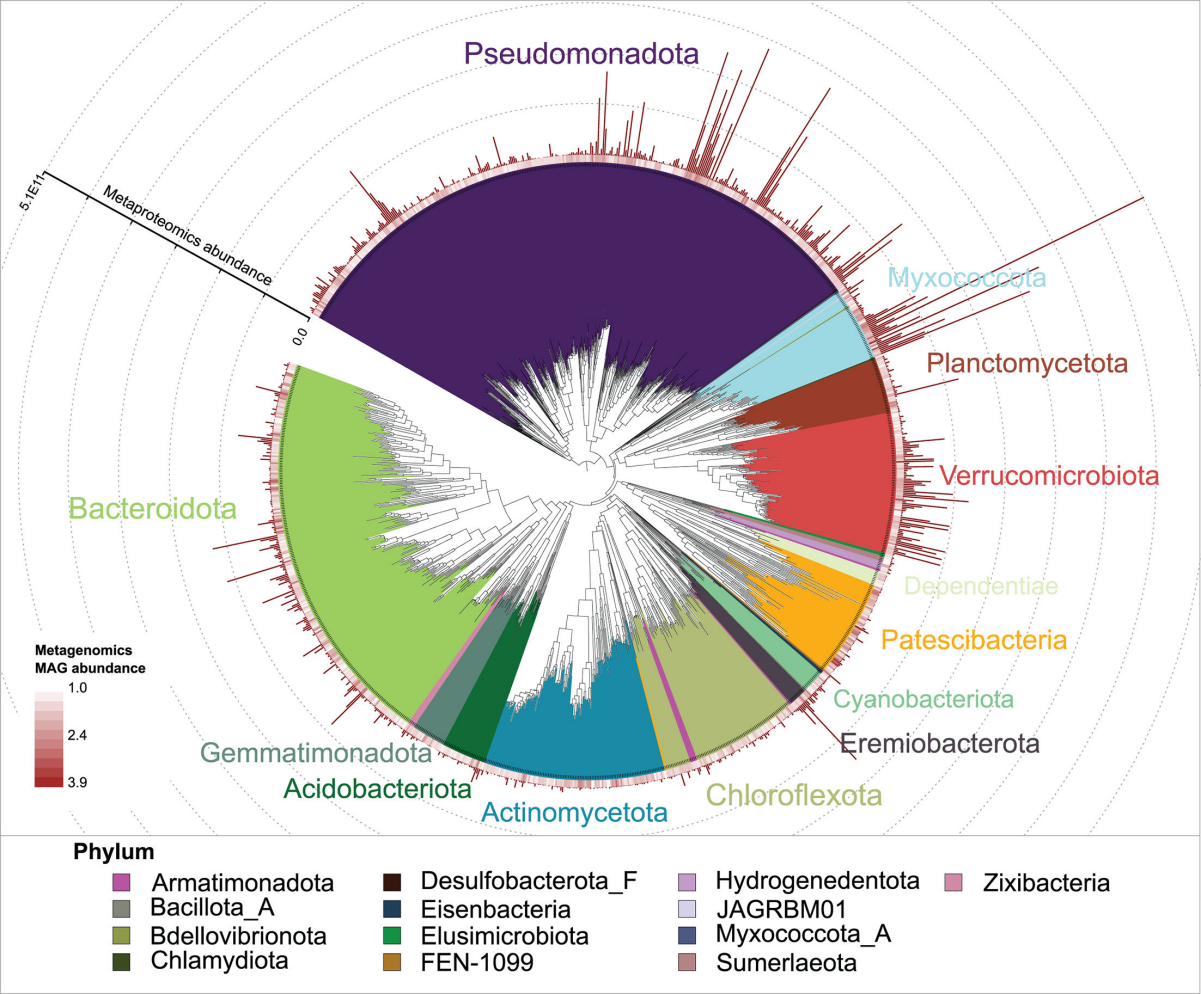
Phylogenetic placement of 1,115 medium-to-high quality MAGs within the lake enrichments. The figure depicts the phylogenetic placement of MAGs in a maximum-likelihood tree (FastTree) annotated with their assigned phylum taxonomy; the most prominent ones are highlighted within the figure, and the remaining ones are beneath. The circular heatmap on the edge shows metagenomic coverage calculated using CoverM, and the bars on the outer circle represent the metaproteomic abundances (LFQ values, Top-N, *n* = 3) summed per MAG, square-root transformed for visualization, and averaged across the ten eutrophic lake enrichments; see “Metaproteomic sample preparation, data acquisition, and analysis” for full details of protein quantification and normalization.

Samples of these enriched cultures were submitted for shotgun metagenomic and metaproteomic analyses. Assembly and binning of the metagenomes resulted in the recovery of 1,331 MAGs plus an additional 31 MAGs from the control lakes, hereafter referred to as CMAGs (Table S4). The 1,331 MAGs contained 60 species unique to the eutrophic lake enrichments, and five species unique to the control lake enrichments (Table S5); 1,115 MAGs were of medium-to-high quality (completeness >50%, contamination <10%) and are presented in Fig. 1. In practice, the actual quality distribution substantially exceeded this threshold with completeness ranging between 75% and 100%, and contamination levels were generally low (Fig. S2). A full summary of MAG quality metrics is provided in Table S4. Using metaproteomics, we detected in total 28,501 expressed proteins across all MAGs and quantified them per lake enrichment (Table S6). Replicate enrichments clustered tightly within lakes in both metagenomic and metaproteomic ordination analyses (Fig. S2), confirming the reproducibility of community composition and protein expression patterns across biological triplicates and supporting the robustness of lake-specific conclusions drawn below.

### A core microbiome was present in all eutrophic lake enrichments

Of the 1,331 MAGs, 181 were detected by metagenomic read mapping in all eutrophic lake enrichments and are hereafter referred to as the core microbial community. These included members of Actinomycetota, Bacteroidota, Chloroflexota, Eremiobacterota, Gemmatimonadota, Patescibacteria, Pseudomonadota, and Verrucomicrobiota, taxa commonly associated with sedimentary environments (52–54). From an ecological perspective, this core community likely represents a set of metabolically versatile and environmentally resilient taxa that persist across systems/lakes due to their central roles in redox cycling and organic matter turnover.

Based on metagenomic reconstruction, the core community could be classified into three major functional groups: (i) non-denitrifiers involved in fermentation and/or β-oxidation, (ii) heterotrophic denitrifiers utilizing fermentation products for growth, and (iii) denitrifiers capable of lignocellulose degradation. This structure is consistent with a putative trophic organization built on inferred metabolic interdependence, where primary fermenters and oxidizers generate low-molecular-weight carbon substrates that sustain denitrifiers. We note that this interpretation is based on endpoint community data and does not constitute direct evidence of temporal stability. Interestingly, and as detailed further in section 3.2, lignocellulose degradation appeared to be largely carried out by species outside the core microbiome, indicating that the identity of key degraders differs between lake enrichments. This pattern suggests a degree of local specialization, possibly reflecting differences in selective pressures imposed during enrichment, which may in turn relate to variation in the quantity and composition of lignocellulosic material along individual shorelines, although this was not directly evaluated. Such a distribution aligns with the core-satellite species hypothesis (55), in which a metabolically coherent core supports ecosystem-level stability, while satellite taxa contribute to local functional diversity and adaptability in response to spatial heterogeneity in organic matter sources.

Group I comprised *Bosea robiniae* (MAG.1128), *Cellulomonas soli* (MAG.0449), and unclassified *Afipia* species (*Afipia* sp017308495; MAG.0395), none of which contained genes for denitrification. These organisms likely contribute to primary organic matter turnover through fermentation and β-oxidation, supplying small intermediates such as organic acids and alcohols to downstream respiratory guilds.

Group II included multiple *Acidovorax* species (*A. delafieldii_B,* MAG.0204, MAG.0329, MAG.0799, MAG.1180, and MAG.1108; *A. radicis_A,* MAG.0878), *Mesorhizobium qingshengii* (MAG.0280), several unclassified *Afipia* species (*Afipia* sp024707075; MAG.0356, MAG.0832, MAG.0681, MAG.0945, MAG.1265, and MAG.1119), as well as unclassified *Phaeospirillum* (MAG.1287), *Thermomonas* (MAG.0911), and *Giesbergeria* (MAG.0026). Many of these MAGs represent heterotrophic denitrifiers with either complete or truncated denitrification gene sets. Genomic profiles indicate capacities for fermentation-linked carbon metabolism, including glycolysis, pyruvate oxidation, acetate assimilation via acetyl-CoA, and fatty acid oxidation, but only limited potential for degradation of complex polysaccharides (as reflected by few CAZymes in the genomes). Ecologically, these organisms likely occupy secondary consumer roles, coupling the oxidation of fermentation products to nitrogen reduction and thereby mediating a key interface between carbon and nitrogen cycling.

Group III consisted exclusively of *Giesbergeria hankyongi* (MAG.0172, MAG.0472, MAG.0875, and MAG.1158), a recognized denitrifier (56). Two MAGs (MAG.0472 and MAG.0875) encoded complete denitrification pathways, while two (MAG.1158 and MAG.0172) were truncated. *G. hankyongi* genomes contained numerous CAZymes, consistent with its previously observed role as a major degrader in denitrifying woodchip bioreactors (57). However, only one CAZyme was detected in the proteomic data set, suggesting either a shift toward utilization of simpler carbon substrates under the conditions studied here, or limited metaproteomics capture due to sample complexity. Thus, while *G. hankyongi* genomically bridges lignocellulose degradation and denitrification, its realized role may vary along environmental or substrate gradients, functionally overlapping with group II under certain conditions.

Beyond these three main groups, several additional core MAGs displayed metabolic versatility spanning multiple biogeochemical cycles. For instance, multiple *Afipia* sp024707075 MAGs, one *G. hankyongi* MAG, and one Sulfuritalea-related MAG encoded genes for both thiosulfate oxidation and complete denitrification, suggesting potential for autotrophic denitrification. Such dual capabilities may confer ecological advantages in redox-fluctuating environments, where access to both organic and inorganic electron donors allows flexible participation in C-, N-, and S-transformations.

**Fig 2:**
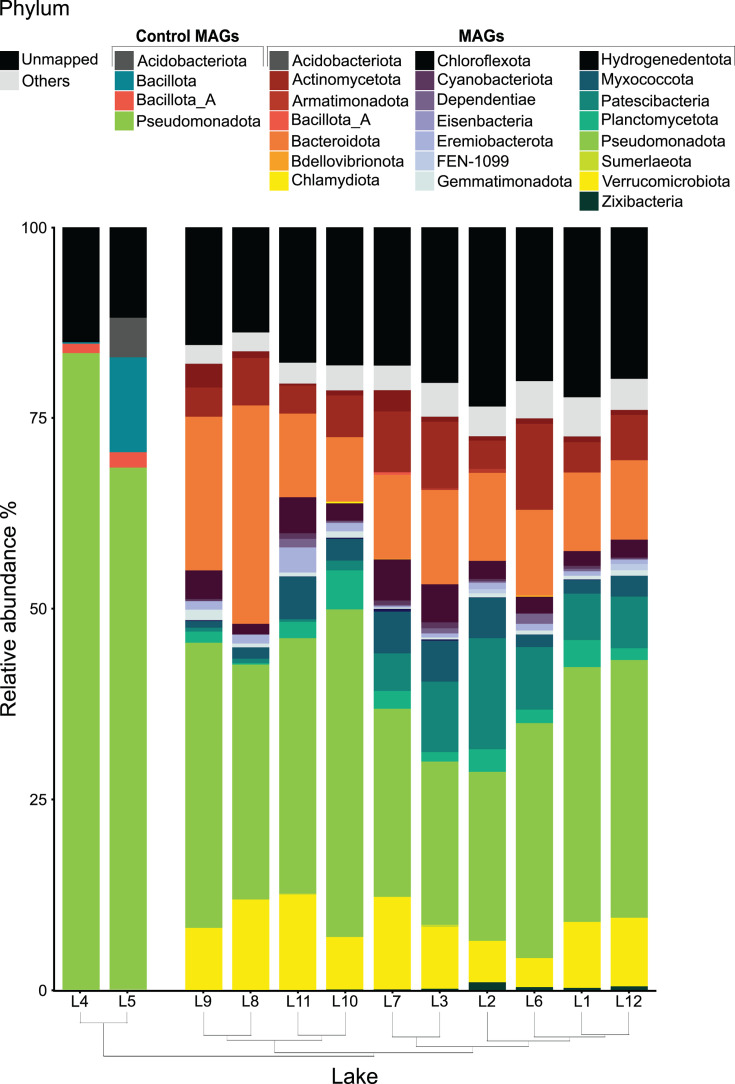
A core microbiome-relative abundance and taxonomic placement of the 200 most abundant MAGs across all lake enrichments. The figure shows the phylum-level distribution of the 200 most abundant MAGs for each lake enrichment. Lake enrichments L4 and L5 are control samples (lakes not exposed to nitrate leaching), while the others originate from eutrophic lakes (Table S1). The hierarchical clustering was analyzed in R with the package pheatmap v.1.0.12.

To illustrate the overall community structure, the phylum-level distribution of the 200 most abundant MAGs is shown in Fig. 2. Notably, lake enrichments L8, L9, L10, and L11 cluster together in community composition (Fig. 2) and also showed comparatively lower cumulative N-gas recovery over the enrichment period (Fig. S1), suggesting a possible link between community structure and denitrification efficiency. The more active lake enrichments (L1, L2, L3, L6, L7, and L12) were characterized by a relatively higher proportion of Patescibacteria—ultrasmall obligate epibionts with highly reduced genomes dependent on host bacteria (58)—possibly reflecting a more abundant and active host community in these enrichments. Furthermore, Fig. 2 highlights the dominance of typical sediment-associated phyla such as Actinomycetota, Bacteroidota, and Pseudomonadota, several of which are also well represented within the core microbiome. The presence of these widespread lineages across the lake enrichments provides a taxonomic scaffold upon which functional differentiation—particularly in lignocellulose degradation—appears to occur.

### Lignocellulose degradation in eutrophic lakes

To better understand how lignocellulose is degraded during denitrification, we interrogated all MAGs for the expression of CAZymes. CAZymes are encoded in the genomes of all detected MAGs; however, when looking at the metaproteomics data for expressed CAZymes (Table S6), only 160 MAGs and 16 CMAGs expressed their CAZymes. From this, we could annotate 609 catalytic CAZy domains and 160 CBMs, corresponding to 77 different enzyme activities targeting lignocellulose (Table 1 and Tables S2 and S3). While comprehensive, this inventory should be considered a conservative estimate, as additional CAZyme-expressing organisms likely exist below the mass spectrometric detection threshold in these complex community samples.

**TABLE 1:** Carbohydrate-active enzymes detected at the protein level that may take part in anaerobic lignocellulose deconstruction^*k*^

Substrate	Predicted enzyme activity	Expressed CAZymes
Cellulose	Cellobiohydrolase, reducing end-acting	GH48, GH48;CBM2, GH48;CBM3
	Cellobiohydrolase, non-reducing end-acting	CBM64;GH6
	Endo-β-1,4-glucanase	GH5,^*a*^ GH5_1;CBM3, GH5_2, GH5_4,^*a*^ GH5_5, GH5_25, GH5_39, GH5_55^*a*^
		GH6, GH6;CBM2, GH8,^*a*^ GH12,^*a*^ GH44,^*a*^ GH51_3, CBM8;CBM8;GH51_3, GH148
	Endo-β-1,4-glucanase, processive	GH9, GH9;CBM3;CBM3, CBM4;GH9, CBM30;GH9
	Exo-β-1,4-glucanase/cellodextrinase^*b*^	GH5_53
	Multifunctional endo-β-1,4-glucanase, exo-β-1,4-glucanase, and cellobiohydrolase	GH12;GH5_53;CBM3;GH6
	Cellodextrin phosphorylase	GH94
	Bifunctional cellodextrin phosphorylase and cyclic β-1,2-glucan synthase	GT84;GH94
	β-Glucosidase, non-reducing end-acting^*b*^	GH3
	Lytic polysaccharide monooxygenase	AA10;CBM2
	FAD-dependent gluco-oligosaccharide oxidase	AA7
	Glucose oxidase/glucose dehydrogenase	AA3_2^*c*^
	Pyrroloquinoline quinone-dependent (glucose/sorbosone) dehydrogenase	AA12
Xyloglucan	(Xyloglucan-specific) endo-β-1,4-glucanase	GH5_4,^*a*^ GH12,^*a*^ GH44^*a*^
	α-Xylosidase	GH31,^*d*^ GH31_3, GH31_4
	β-Galactosidase, terminal non-reducing	GH2,^*e*^ GH35^*e*^
	α-L-Fucosidase	GH29,^*e*^ GH29;CBM32, GH95,^*e*^ GH95;CBM35^*e*^
	Acetylesterase	CE20
Mixed-linkage β-glucan	Endo-β-1,3-glucanase	CBM56;GH64, CBM38;GH81;CBM6, GH128
	Endo-β-1,4-glucanase^*a*^	See footnote
	Exo-β-1,4-glucanase/cellodextrinase^*b*^	See footnote
	β-Glucosidase, non-reducing end-acting^*b*^	See footnote
Laminarin (brown algae)	Glucan 1,3-β-glucosidase	GH55_1
(Galacto)(gluco)mannan	Endo-β-1,4-mannanase	GH5_8, GH5_8;CBM13, GH5_10, GH5_17, GH5_55,^*a*^ GH26, GH26;CBM23;CBM23
		CBM35;GH26;CBM46;CBM3, CBM35;GH26;CBM46;CBM46;CBM3
		CBM35;CBM35;CBM35;GH26, CBM54;GH26;CBM27;CBM23, CBM54;GH26;CBM23;CBM59
		CBM54;GH26;CBM27;CBM23;CBM59, GH134
	β-Mannosidase, terminal non-reducing	GH2
	β-Glucosidase, terminal non-reducing^*b*^	See footnote
	β-1,4-Mannooligosaccharide phosphorylase	GH130_2
	β-1,4-Mannosylglucose phosphorylase	GH130_1
	β-Mannoside phosphorylase	GH130_11
	α-Galactosidase	GH27,^*f*^ GH36^*f*^
Xylan	Endo-β-1,4-xylanase	GH5_21;GH5_35, GH10, GH10;CBM2, GH10;CBM6;CBM22;CBM22
		GH10;CBM6;CBM22;CBM22;CBM22, GH10;CBM22;CBM22;CBM22
		CBM22;CBM22;GH10;CBM9, CBM22;CBM22;GH10;CBM9;CBM22;GH10;CBM9
		CBM85;GH10, GH11, GH30^*g*^
	(Glucuronoxylan) endo-β-1,4-xylanase	GH30_8, GH30_8;CBM35
	β-1,4-Xylosidase	GH30,^*g*^ GH30_2, GH52
	Acetylxylan esterase	CE2, CE3, CE4,^*h*^ CE4;CBM36,^*h*^ CE6, CE7
	Acetylxylan esterase/feruloyl esterase	CE1
	Bifunctional acetylxylan esterase and polysaccharide synthase	CE4;GT2^*h*^
	α-L-arabinofuranosidase, non-reducing end-active	GH43_16;CBM6, GH51_1,^*i*^ GH54,^*i*^ GH62^*i*^
(Glucurono)-xylan	α-Glucuronidase	GH4,^*j*^ GH67, GH115
Lignin–carbohydrate complexes	(4-*O*-Methyl-)glucuronate–lignin esterase	CE15
Pectin	Pectate lyase	PL1;CBM77, PL1_6, PL3_1, PL9
	Endo-polygalacturonase	GH28
	Rhamnogalacturonan lyase	PL11, PL9
	Bifunctional rhamnogalacturonan endolyase and pectin acetylesterase	PL11;CE12;CE12
	Exo-poly-α-digalacturonosidase, non-reducing end-active	GH28
	Pectate disaccharide-lyase (exopolygalacturonate lyase); reducing end-active	PL22
	Oligogalacturonate lyase	PL22
	D-4,5-unsaturated α-galacturonidase (unsaturated rhamnogalacturonyl hydrolase)	GH105
	Pectin methylesterase	CE8
	Pectin acetylesterase	CE12
	Endo-β-1,2-apiosidase	GH140
	Endo-β-1,4-galactanase	CBM61;GH53
	β-Galactosidase, terminal non-reducing	GH35^*e*^
	β-L-arabinofuranosidase	GH27^*f*^
		GH36^*f*^
	α-L-arabinofuranosidase, non-reducing end-active	GH51_1,^*i*^ GH54,^*i*^ GH62^*i*^
Lignin	Laccase	AA1
	GMC oxidoreductase	AA3
	Aryl alcohol oxidase/aryl alcohol dehydrogenase	AA3_2^*c*^
	Vanillyl-alcohol oxidase	AA4
	NADPH:*p*-benzoquinone oxidoreductase	AA6
Starch	α-Amylase	GH13_1, GH13_6, GH13_19
	α-Amylase/neopullulanase	GH13_46
	Isoamylase (debranching enzyme)	GH13_11
	Pullulanase (limit dextrinase)	GH13_13;GH13_13, CBM41;GH13_13
	Bifunctional α-amylase and pullulanase	CBM41;CBM41;GH13_12;GH13_41
	Glucoamylase (terminal non-reducing ends of starch polysaccharides)	GH15
	α-1,4-Glucan phosphorylase	GT35
	α-Glucosidase (terminal non-reducing ends of malto-oligosaccharides)	GH13_38, GH31,^*d*^ GH97
	Maltose-6′-phosphate glucosidase; α,α-trehalose-6-phosphate glucosidase	GH4^*j*^
	4-α-Glucanotransferase (dextrin glycosyltransferase)	GH77
	Starch synthase (maltosyl transferring)	GH13_3
	Glycogen synthase	GT5
	α-1,4-Glucan branching enzyme [amylo-(1,4→1,6)-transglycosylase]	GH13_9, CBM48;GH13_9
	Cyclomaltodextrin glucanotransferase	GH13_2;CBM20
	Malto-oligosyltrehalose synthase	GH13_26
	Malto-oligosyltrehalose trehalohydrolase	GH13_10
	α,α-Trehalase	GH37
	Trehalose-6-P synthase	GT20

^*a*^ Some enzyme classes with endo-β-1,4-glucanase activity may exhibit activity toward substrates other than cellulose, including xyloglucans, xylans, mannans, and/or chitosan. Corresponding CAZyme domains and MAGs are listed at cellulose.

^*b*^ Some exo-β-1,4-glucanases and β-glucosidases also hydrolyze β-(1→4)-linked D-glucosyl residues from the nonreducing end of oligosaccharides from mixed-linkage β-glucan and glucomannan. Corresponding CAZyme domains and MAGs are listed at cellulose.

^*c*^ Some AA3_2 enzymes listed under aryl alcohol oxidases or dehydrogenases may be glucose oxidases or dehydrogenases.

^*d*^ GH31 enzymes hydrolyze α-xylosides in xyloglucan, α-glucosides in starch, or α-*N*-acetylgalactosaminides in O-glycans. Corresponding MAGs are listed at xyloglucan.

^*e*^ α-L-fucosidases and β-galactosidases hydrolyze α-L-fucosyl and β-D-galactosyl substitutions, which are common in xyloglucan, in O-glycans of proteins, and the latter one also in pectic galactan. Corresponding MAGs are listed at xyloglucan.

^*f*^ Some GH27 and GH36 enzymes listed under α-galactosidases at galacto(gluco)mannan may be β-L-arabinosidases hydrolyzing pectic arabinan and arabinogalactan. Corresponding MAGs are listed at galacto(gluco)mannan.

^*g*^ HMMer annotation does not specify subfamily; some GH30 members listed under endo-β-1,4-xylanases may be β-xylosidases or endo-β-1,6-glucanases.

^*h*^ CE4 enzymes deacetylate xylosyl residues in acetylxylan or *N*-acetylglucosamine residues in peptidoglycan, chitin, or chito-oligosaccharides. Corresponding MAGs are listed at xylan.

^*i*^ α-L-arabinofuranosidases hydrolyze the terminal arabinosyl substitutions in arabinoxylan, arabinan, and arabinogalactan. Corresponding MAGs are listed at xylan.

^*j*^ GH4 enzymes include maltose-6′-phosphate glucosidases, α-glucuronidases, α-galactosidases, 6-phospho-β-glucosidases, or α-glucosidases. Only the first two (most prevalent) activities are indicated in this table. Corresponding MAGs are listed at starch.

^*k*^ The table organizes the detected CAZymes based on their predicted activity and target substrate and provides the identified CAZy modules in the domain structure. The MAGs expressing these proteins are listed in Table S2; 77 different enzyme activities targeting lignocellulose are shown. Domains belonging to the same protein are separated by a semicolon. In addition, proteins of unknown function carrying CBM47, CBM44, CBM26, or CBM57 (i.e., without identification of catalytic CAZyme modules) were detected; these proteins are not listed in the table. Abbreviations: AA, auxiliary activity; CBM, carbohydrate-binding module; GH, glycoside hydrolase; GT, glycoside transferase; PL, polysaccharide lyase; CE, carbohydrate esterase.

**Fig 3:**
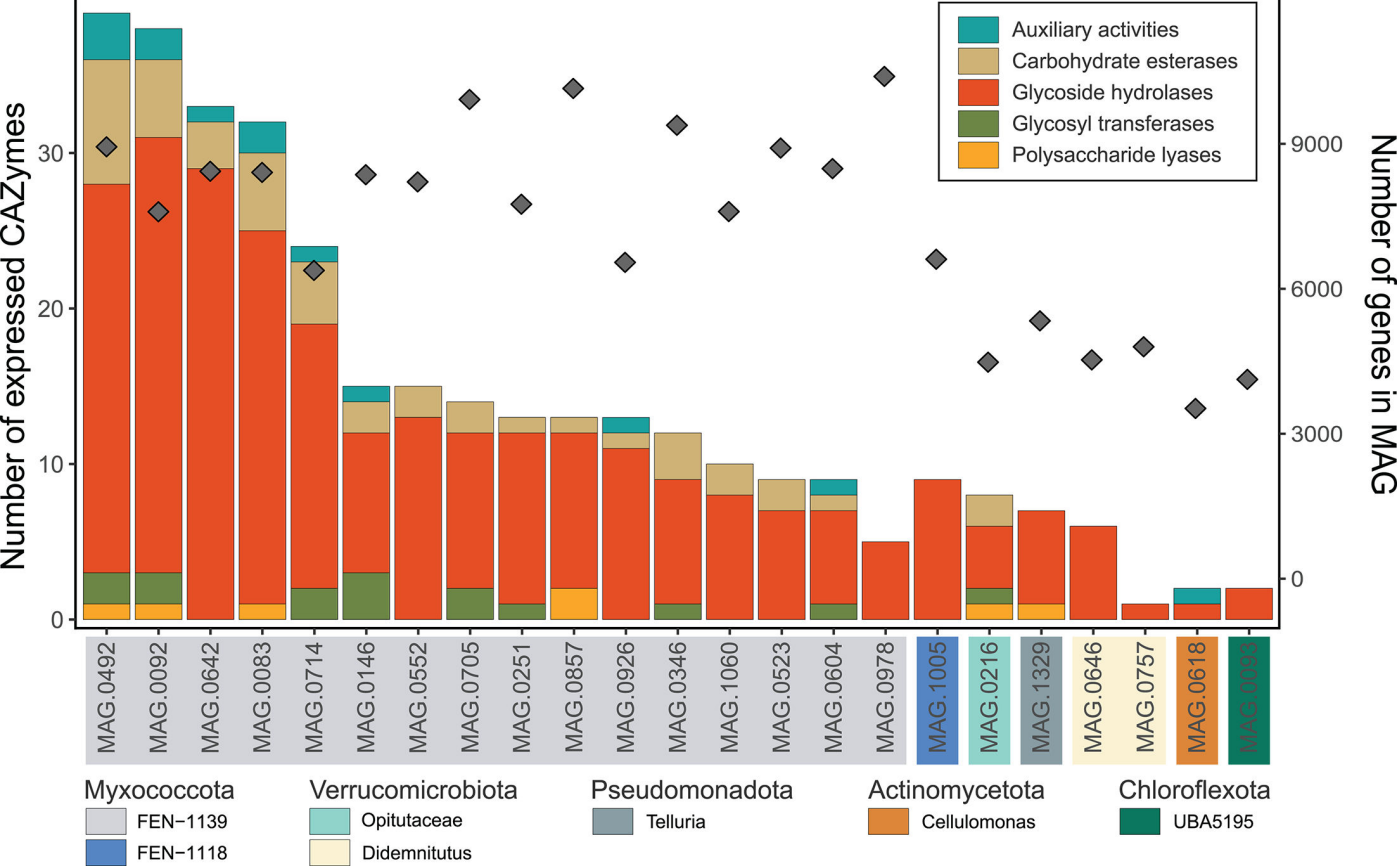
The key lignocellulose-degraders. The figure shows the MAGs with the most CAZymes expressed within the eutrophic lake enrichments, as well as the distribution of CAZyme families per MAG. The gray diamonds show the predicted gene count per MAG for assessment of CAZyme richness.

The MAGs expressing the most CAZymes targeting lignocellulose were assigned to gram-negative FEN-1139, FEN-1118, *Didemnitutus*, *Opitutaceae*, *Telluria*, and UBA5195 species, and gram-positive *Cellulomonas gelida* (Fig. 3), while the dominating CMAGs belonged to gram-negative *Paenibacillus* species and *Paenibacillus silagei*, *Serratia_A fonticola*, and *Paludibacterium* species.

The observation that the top CAZyme-expressing MAGs in our lignocellulose enrichments map to FEN-1139 and FEN-1118—genera with little prior characterization—highlights the potential for yet-undiscovered microbial actors in sedimentary polymer decomposition. Within FEN-1139, different MAGs dominate distinct lake enrichments: MAG.0492 is predominant in sample L11, MAG.0092 in L2 and L7, and MAG.0642 in L2, etc. This pattern emphasizes that the identity of the key lignocellulose degrader varies between lake enrichments; however, FEN-1139 remains a consistently important lineage, serving as a dominant genus placeholder within the Polyangiaceae family.

In general, in both MAGs and CMAGs, we detected CAZymes that target the lignocellulose subfractions cellulose, xyloglucan, pectin, xylan, mannan, starch, as well as protein glycosylations, which together contribute to the decomposition of plant cell walls (Table 1 and Table S2). In addition, we found enzymes that can cleave peptidoglycan, which is present in bacterial cell walls, and enzymes that can cleave chitin, which is present in the exoskeleton of fallen arthropods and in fungal cell walls (Table S3). The presence of oxidoreductases belonging to various AA families in the CAZy database is indicative of the lignin-modifying potential of the microbial community. This is detailed further below.

#### Cellulose

We detected extended sets of cellulose-degrading machineries expressed by 12 MAGs: MAG.0092 (16 proteins), MAG.0642 (15), MAG.0492 (9), MAG.0714 (9), MAG.0083 (8), MAG.0552 (7), MAG.0857 (7), MAG.0346 (6), MAG.0926 (6), MAG.0146 (5), MAG.0251 (5), and MAG.1060 (5); all assigned to FEN-1139. In MAG.0092, the wide variety of cellulose-active CAZymes included GH8, GH12, and GH44 endoglucanases; GH9 processive endoglucanases (single-domain and multi-domain linked to family 3, 4, or 30 CBM), and a GH48 cellobiohydrolase, which releases cello-oligosaccharides, and a GH3 β-glucosidase, which depolymerizes the cello-oligosaccharides to glucose. MAG.0642, on the other hand, expressed a different variety of endoglucanases (GH5_2, GH5_25, GH6, GH8, and GH12), one CBM4-linked GH9 processive endoglucanase, no cellobiohydrolase, and a GH3 β-glucosidase. Alternatively, after depolymerizing cellulose using GH5_2, GH_5, GH6, GH8, GH12, and GH44 endoglucanases and two processive GH9 endoglucanases, MAG.0714 (*Opitutaceae*) utilized cellulose as a carbon source through the phosphorolytic pathway, relying on a GH94 cellodextrin phosphorylase instead of a β-glucosidase (59). MAG.0093 (UBA5195) expressed only two enzymes (both cellulose-active): a multifunctional cellulase with an N-terminal GH5_53 endo-β-1,4-glucanase, a C-terminal cellobiohydrolase (CBM3 followed by GH6), and a central GH5_53 exo-β-1,4-glucanase domain, as well as a GH6 cellobiohydrolase with an N-terminal CBM64. Furthermore, MAG.0618 (*C. gelida*) secreted an AA10 LPMO with a C-terminal CBM2, capable of oxidatively cleaving crystalline cellulose in an endo fashion, which was co-expressed with a GH48 cellobiohydrolase (also carrying a C-terminal CBM2). Interestingly, in our previous work on denitrifying bacteria growing on woodchips, we also observed this LPMO expressed in anoxia (57), and indeed, biochemical characterization of this enzyme confirmed its functional requirements for O_2_ or H_2_O_2_ (60).

#### Xyloglucan

In addition to cellulose, many of the endo-β-1,4-glucanases are likely to be able to cleave the polysaccharide backbone of xyloglucan (GH5_4, GH12, and GH44), or sometimes even xylan (GH8), glucomannan (GH5), and chitosan (GH8). Regarding xyloglucan degradation, we could not identify a single MAG that would co-express enzymes cleaving both the β-(1→4)-glucan main chain and either the α-(1→6)-xylosyl, β-(1→2)-galactosyl, α-(1→2)-fucosyl, or acetyl substitutions. Although we detected a GH31_4 α-xylosidase expressed together with a GH35 β-galactosidase by MAG.0524, most xyloglucan-debranching enzymes, including a GH31_4 α-xylosidase by MAG.1146, GH2 β-galactosidases by MAG.0007, MAG.0328, and MAG.0830, a GH29 α-L-fucosidase by MAG.0821, GH95 α-L-fucosidases by MAG.0163, MAG.0279, and MAG.0327, and a CE20 acetylesterase by MAG.0393 were detected as sole proteins expressed by the corresponding MAGs. The distribution of xyloglucan-debranching activities across multiple MAGs, with no single MAG encoding a complete degradation pathway, is consistent with community-level cooperative degradation—a pattern mirroring the broader metabolic interdependence observed across functional guilds in these enrichments. We note, however, that apparent pathway incompleteness within individual MAGs may also reflect incomplete genome recovery during assembly, and therefore, the absence of evidence should not be interpreted as evidence of absence.

#### Xylan

Several MAGs expressed extensive enzyme sets for the decomposition of xylans, including acetylated and feruloylated glucuronoarabinoxylan typically occurring in grasses. The main MAGs with the highest number of xylan-active CAZymes include four MAGs annotated as FEN-1139, namely MAG.0492 (20 proteins), MAG.0083 (17), MAG.0092 (14), and MAG.0642 (14). Of these, MAG.0492 expressed GH10, GH11, and GH30 endo-β-1,4-xylanases, GH30 and GH30_2 β-xylosidases, GH43_16 and GH54 α-L-arabinofuranosidases, CE2 and CE3 acetylxylan esterases, CE1 acetylxylan or feruloyl esterases, and four CE15 (4-*O*-methyl-)glucuronate–lignin esterases. In addition to these enzyme types, the other three xylan-decomposing MAGs expressed GH52 β-xylosidase (MAG.0092), GH62 α-L-arabinofuranosidases (MAG.0083, MAG.0092, and MAG.0642), GH67 α-glucuronidases (MAG.0092 and MAG.0642), and CE6 acetylxylan esterases (MAG.0092 and MAG.0642). Interestingly, all four MAGs expressed enzymes capable of cleaving next to (4-*O*-methyl)-glucuronoyl [GH67 α-glucuronidases and CE15 (4-*O*-methyl-)glucuronate–lignin esterases] and feruloyl units (CE1 feruloyl esterases), both of which can form bridges between xylan and lignin in plant cell walls.

#### (Galacto)(gluco)mannan

Regarding decomposition of (galacto)(gluco)mannan, the main species included MAG.0216, MAG.0497, MAG.0646, and MAG.1192 (all belonging to the Opitutaceae family), which co-expressed combinations of enzymes cleaving the glucomannan backbone into oligosaccharides and monomers, as well as enzymes cleaving galactosyl or acetyl substitutions. MAG.0646 expressed a GH26 endo-β-1,4-mannase, a GH130_2 β-1,4-mannooligosaccharide phosphorylase, a GH130_1 β-1,4-mannoglucose phosphorylase, and a GH27 α-galactosidase. On the other hand, we detected a more complete set of enzymes by MAG.0216, including a GH5_8 endo-β-1,4-mannase (with a C-terminal CBM13), a GH51_3 endo-β-1,4-glucanase (with two N-terminal CBM8 domains), a GH130_1 β-1,4-mannoglucose phosphorylase, a GH27 α-galactosidase, and a CE2 acetyl esterase. In the case of MAG.1192, we detected only three CAZymes, all taking part of galactomannan depolymerization: a GH26 endo-β-1,4-mannase, a GH130_2 β-1,4-mannooligosaccharide phosphorylase, and a GH27 α-galactosidase. On the other hand, for MAG.0642, expressing the most diverse enzymes in terms of substrate specificity, we detected three types of endo-β-1,4-mannanases (GH5_10, GH26, and GH134) and no debranching enzymes.

#### Pectin

Concerning pectin degradation, we detected 52 pectin-active enzymes expressed by 33 MAGs (most MAGs expressing one or two pectin-active enzymes only) and identified over 20 different CAZy domain structures. Of these, MAG.0492 (5 proteins), MAG.0083 (4), MAG.0146 (3), MAG.0714 (3), and MAG.1329 (*Telluria*) (3) expressed more than two proteins. The high variety of pectin-degrading enzymes is not surprising, considering the complex structure of pectin, including rhamnogalacturonan types I (RG-I; rhamnogalacturonan backbone with alternating α-D-galacturonic acid and α-L-rhamnose residues carrying arabinan and (arabino)galactan side chains) and II (RG-II; homogalacturonan backbone carrying shorter side chains of varying composition). Expressed by MAG.1329, we detected a CE8 pectin methylesterase (demethylating the polysaccharide backbone of RG-II) and a PL1_6 pectate lyase (depolymerizing the (demethylated) polysaccharide backbone of RG-II). By MAG.0083, we detected a PL9, which is either a pectate lyase or a rhamnogalacturonan lyase (depolymerizing the polysaccharide backbone of RG-II or RG-I, respectively), a GH105 unsaturated rhamnogalacturonyl hydrolase, which cleaves unsaturated rhamnogalacturonan dimers (disaccharides released from RG-I by the action of rhamnogalacturonan lyases) into unsaturated D-galacturonic acid and L-rhamnose monomers, and two GH62 α-L-arabinofuranosidases, which cleave the arabinan (and arabinogalactan) side chains of RG-I. Furthermore, we identified enzymes expressed by MAG.0492 (FEN-1139), one of the most prevalent MAGs in terms of the total number of proteins expressed and the types of substrates targeted (Fig. 3), which targeted both RG-I and RG-II. These enzymes included a PL9 pectate lyase (RG-II-active) or rhamnogalacturonan lyase (RG-I), a GH28 endo- or exo-polygalacturonase (RG-II), a GH105 unsaturated rhamnogalacturonyl hydrolase (RG-I), and two GH54 α-L-arabinofuranosidases (RG-I).

#### Starch

In addition to the plant cell wall polysaccharides, we identified enzymes targeting the storage polysaccharide starch. Although starch is built up of a single building block, α-D-glucose, we identified 17 distinct CAZy domains (including subfamilies) expressed by 28 MAGs, emphasizing the complexity of starch structure and the importance of starch as a carbon source for life. MAG.1329 expressed the highest detected number of starch-active enzymes including GH13_6 and a GH13_46 α-amylases, cleaving α-(1→4)-linked D-glucan chains randomly in an endo fashion, a GH13_13 pullulanase with N-terminal CBM41, cleaving α-(1→6)-linked branching points in amylopectin, and a GH31 α-glucosidase, cleaving malto-oligosaccharides into glucose. Similarly to cellulose degradation, we detected five GT35 α-1,4-glucan phosphorylases (catalyzing the phosphorolytic cleavage of the terminal α-(1→4)-D-glucosyl units from the non-reducing ends, releasing α-D-glucose-1-phosphate) expressed by five MAGs and a GH4 maltose-6′-phosphate glucosidase (cleaving α-maltose 6′-phosphate into D-glucose and D-glucose-6-phosphate) by one MAG. Of these MAGs, only MAG.0714 expressed also a GH94 cellodextrin phosphorylase in addition to the GT35 α-1,4-glucan phosphorylases.

#### Lignin

Despite our strict maintenance of anaerobic conditions throughout the experiment, several carbohydrate and lignin-active oxidoreductases requiring O_2_ or H_2_O_2_ as a co-substrate for their catalysis were detected. Of the 160 MAGs found in eutrophic lake enrichments to express CAZymes, 35 different MAGs expressed in total 43 AAs, which could indicate potential oxidative degradation of lignocellulosic biomass, in particular cellulose and lignin (Table S2). However, the role of AAs was likely not dominant, as most of the AA enzymes were detected as the sole proteins expressed by their MAGs, with a few exceptions. MAG.0495 (belonging to the family Rhodocyclaceae) expressed four AAs (one AA3_2 GMC oxidoreductase, which could likely be an aryl alcohol oxidase/aryl alcohol dehydrogenase, and three AA4 vanillyl-alcohol oxidases), while no other CAZymes were detected by this species. On the other hand, MAG.0353 (belonging to the family Burkholderiaceae) expressed an AA3 GMC oxidoreductase, which could potentially be a glucose oxidase/dehydrogenase, and a FAD-dependent AA7 gluco-oligosaccharide oxidase, and no other CAZymes. Several MAGs with high numbers of expressed CAZymes (MAG.0083, MAG.0092, MAG.0146, MAG.0492, and MAG.0642; all attributed to the FEN-1139 genus) expressed at least one of three AAs: AA3 GMC oxidoreductase, AA6 NADPH:*p*-benzoquinone oxidoreductase, and AA12 pyrroloquinoline quinone-dependent sugar dehydrogenase—in addition to their arsenal of cellulolytic, xylanolytic, glucomannanolytic, and pectinolytic CAZymes.

Similar AA enzymes for anaerobic lignocellulose degradation have been detected and expressed in previous studies in anoxia (57) and also suggested to play a role in Fenton chemistry (61). The source of O_2_ for these AA enzymes, if active under these conditions, remains unclear. Possible explanations include co-regulation with glycoside hydrolases, roles in Fenton chemistry, or activity at transiently oxic microniches. The data set also contains 66 NOD sequences (see chapter 3.4), and while NOD-derived O_2_ is one speculative possibility, there is no direct evidence for this in the current data set, and purely hydrolytic degradation without oxidative support remains a viable alternative, as demonstrated in fungi (62). Although the detection of expressed oxidoreductases under strict anaerobic conditions is intriguing, the presence of these enzymes may not necessarily indicate oxidative plant cell wall decomposition. Further research is needed to determine their functionality under denitrifying conditions (as studied here) or whether their transcription is merely co-regulated by common inducers of glycoside hydrolase expression.

#### Lignocellulose degradation in enrichments from the control lakes

Regarding the control lake enrichments, expressed CAZymes were also detected within the CMAGs (Table 1 and Table S2) with similar activities as those from the MAGs found in the eutrophic lake enrichments. The dominating species in the controls were *Paenibacillus* sp. (CMAG.01 and CMAG.04), *Paenibacillus silage* (CMAG.21), and *Serratia_A fonticola* (CMAG.11), with CMAG.01 and CMAG.21 being the predominant cellulose, xylan, and glucomannan degraders, and CMAG.11 being the predominant starch degrader with six different expressed enzyme activities. Overall, the detected enzymes correspond to the known diverse metabolic activities of *Paenibacillus* species, including the degradation of starch, xylans, cellulose, and pectin, as well as utilization of lignin (63, 64). CMAG.11 also expressed three enzymes active on peptidoglycan, including a GH23 lysozyme, a GT51 peptidoglycan glycosyltransferase, and a CE9 *N*-acetylglucosamine-6-phosphate deacetylase. Of note, CAZymes targeting polysaccharides present in bacterial or fungal cell walls, mucopolysaccharides, or glycoproteins were expressed in both samples originating from eutrophic and control lakes (Table S3).

### Coupled C- and N-turnover in eutrophic lake enrichments

**Fig 4:**
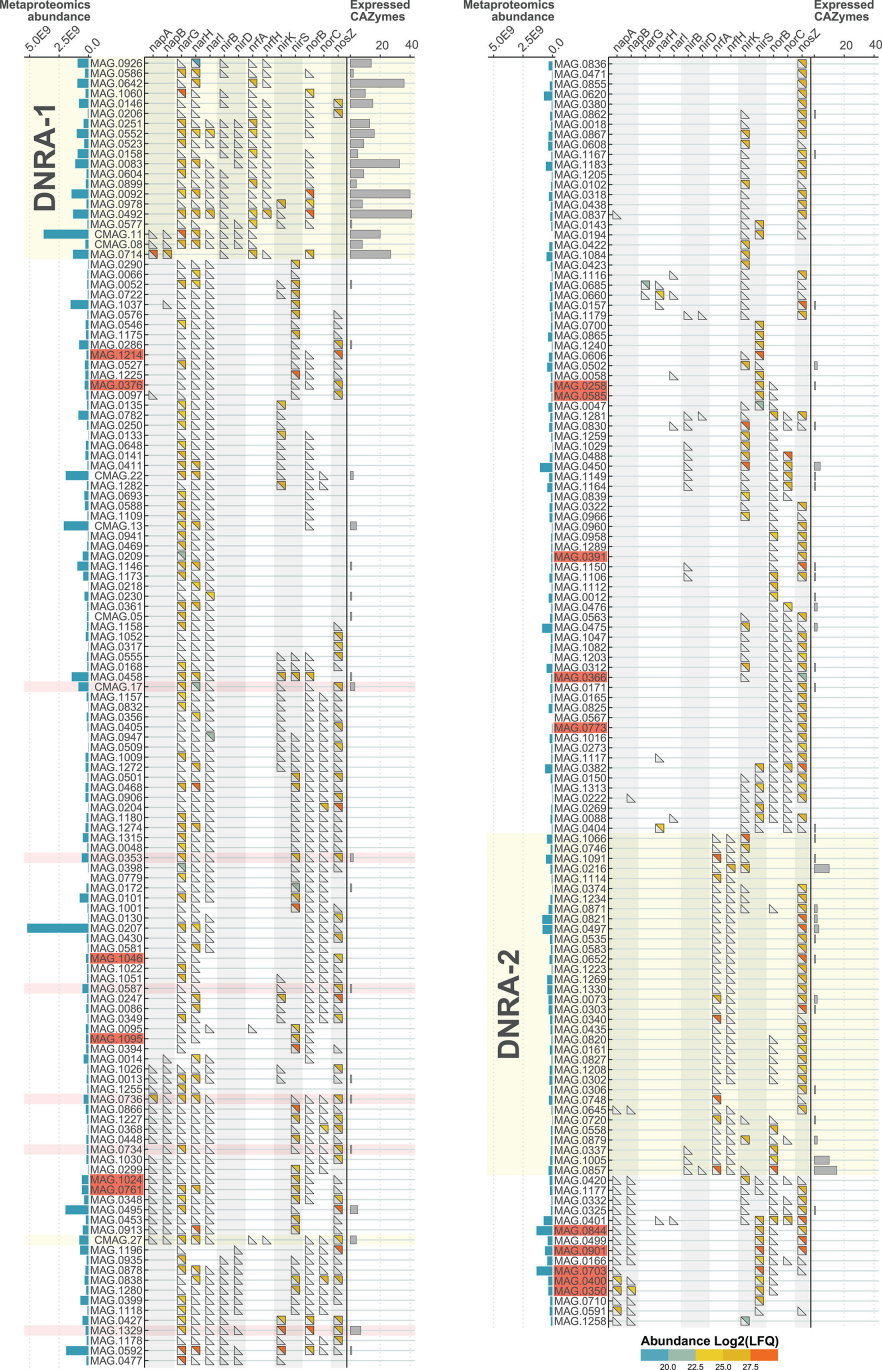
Coupled C- and N-transformations. The figure shows 256 MAGs with at least one expressed enzyme involved in denitrification or DNRA, and the number of expressed CAZymes. Gray triangles indicate whether a gene is present in a MAG’s genome, and colored triangles indicate the proteomic abundance (averaged across the 10 eutrophic lake enrichments) when expressed. The rows are hierarchically clustered to group MAGs with similar gene repertoire. To the left, the total MAG protein abundance is shown (blue bar), and to the right, the number of expressed CAZymes (gray bar) for each MAG. Two clusters of MAGs utilizing DNRA are highlighted in yellow. Full-fledged denitrifiers that also express CAZymes are highlighted as light red rows. MAGs where the *norB* potentially is a *nod* (H-to-N mutation in the active site), see chapter 3.4, have been given a red MAG-name. Note: Due to high sequence homology between *nar* and *nxr* subunits, these may be co-annotated; however, the anoxic, nitrate-amended conditions suggest a functional role in nitrate reduction rather than oxidation.

Looking at MAGs involved in N-metabolism among the 1,331 MAGs and focusing on MAGs that express at least one component of denitrification or DNRA, we find 256 MAGs (Fig. 4). Of these, most MAGs show expression of only a few of the enzyme components present in the genome of the individual MAGs. Given the massive size of this data set and complexity within the samples for proteomics, it is not unlikely that many more enzymes are in fact expressed within the community but either below the mass spectrometer’s detection limit or simply not selected for fragmentation due to too high peptide complexity. Therefore, we do not require expression of all enzyme subunits (e.g., both NorB and NorC) as a filtering criterion for determining whether an active enzyme is present. Instead, we include enzymes with at least one subunit expressed, under the assumption that the remaining subunits are also present but escaped detection.

Regarding denitrification, the conversion of NO_3_^−^ to NO_2_^−^ via Nar is dominant, being expressed in 82 MAGs, whereas Nap occurs in only five MAGs. The reductases converting NO_2_^−^ to NO, NirK and NirS, are both prevalent, found in 30 and 49 MAGs, respectively. Conversion of NO to N_2_O by Nor is detected at the protein level in 28 MAGs, despite being a membrane protein and typically difficult to detect by proteomics. The potential for conversion of N_2_O to N_2_ by NosZ is detected in 108 MAGs, five of which lack other denitrification genes and therefore function as pure N_2_O sinks. Regarding DNRA, 16 MAGs express Nrf, typically in combination with Nar, while some do not have *nar* or *nap* in their genome at all. Surprisingly, NirBD is not expressed in any of the MAGs.

Ecologically, it is fair to assume that what we observe at the community-level is lignocellulose-driven N-metabolism, where electrons flow from the degradation of complex plant polymers in lignocellulose to nitrogen oxides and respiration in denitrification and DNRA. Multiple MAGs across various functional guilds are involved in the process, including primary degraders transforming lignocellulose, and secondary consumers and denitrifiers connected via cross-feeding and syntrophy. As can be seen from Fig. 4, most MAGs involved in N-metabolism do not express any CAZymes, which is in line with the notion that most denitrifiers prefer simple carbons. However, a few key organisms seem to possess a dual capacity for coupled C- and N-transformation. This seems to be most prevalent in DNRA microorganisms (Fig. 4, yellow clusters) where up to 40 CAZymes are being co-expressed with Nar and Nrf in FEN-1139 (MAG.0492), potentially indicating a metabolic shortcut or self-sufficiency, reducing reliance on cross-feeding (cellulolytic to fermentative to denitrifying) and streamlining electron flow from polymer to ammonium. Only very few full-fledged denitrifiers expressed CAZymes (Fig. 4, light red) exemplified by an uncultured *Telluria* species (MAG.1329) expressing seven CAZymes targeting non-cellulosic polysaccharides. Polysaccharide degradation in *Telluria* has been reported previously, including the degradation of xylan and starch (65).

### NO dismutases in the lake enrichments

Since the discovery of putative NODs in *Methylomirabilota* (NC10) bacteria 15 years ago (66, 67), *nod* genes have been identified in many different habitats and species (68–72). Out of the 1,362 MAGs detected in this work, 66 contained a *nod* gene. All of them were identified in the eutrophic lakes, which is not surprising, given the fact that NODs are proposed to convert NO into molecular oxygen and dinitrogen. NO is produced by NirK and NirS during denitrification, but even low concentrations of NO are usually quickly reduced to nitrous oxide (N_2_O) by Nor to avoid cytotoxic effects (73). If NODs convert NO to O_2_ and N_2_, which would circumvent the production of the potent greenhouse gas N_2_O and could potentially lead to reduced emissions both in natural habitats and during biotechnological processes such as the treatment of nitrogen-polluted water (74). However, while there are several publications showing indications for NO dismutation by NOD-containing organisms (67, 75), also some opposing results exist (76, 77), and the NOD enzyme has still not been isolated and characterized biochemically.

**Fig 5:**
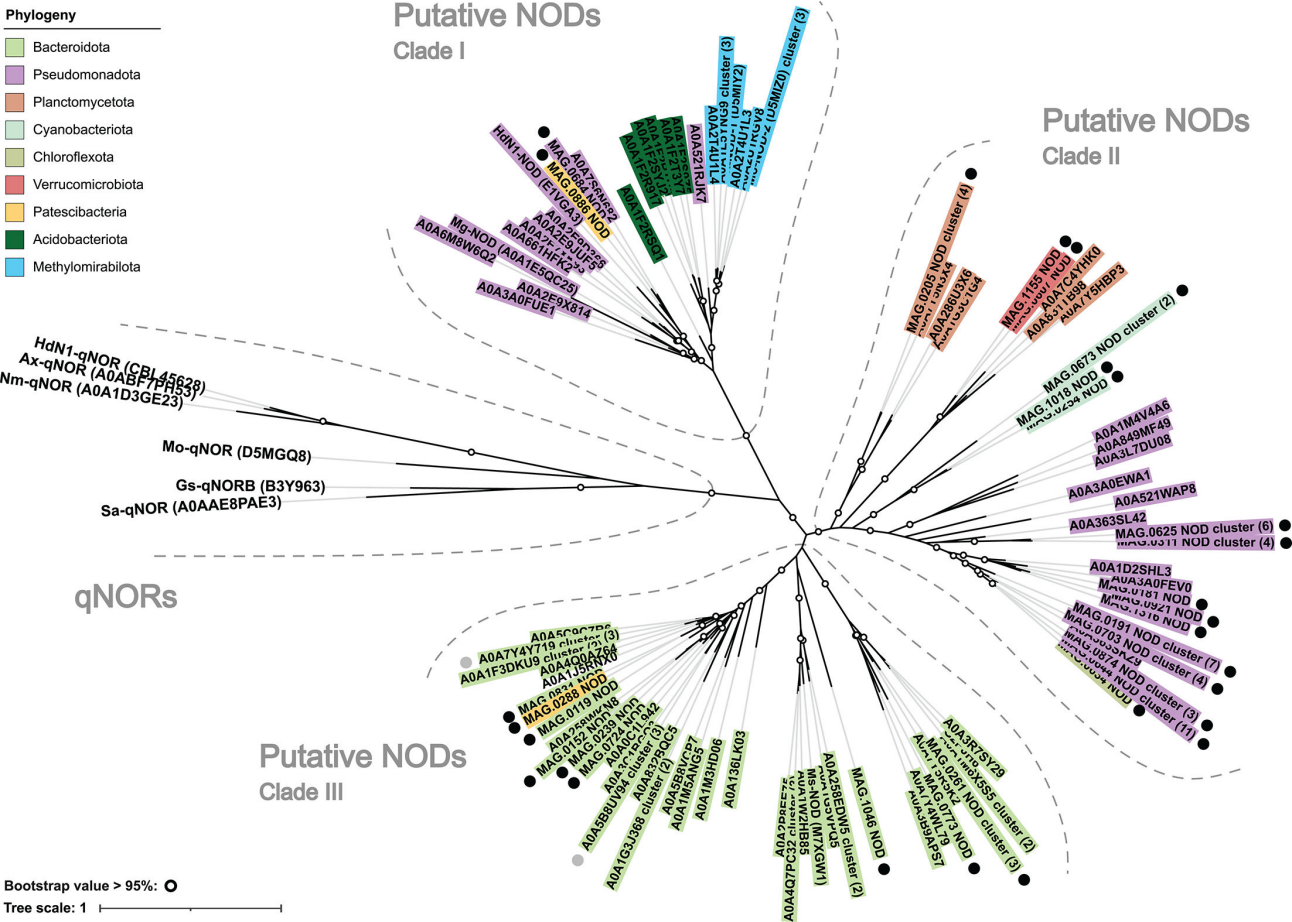
Phylogenetic tree of NODs from the lake enrichments. The figure shows a phylogenetic placement of our 66 NODs together with NODs and qNORs identified from UniProt (accession number given). To reduce redundancy, sequences with >95% identity were clustered, and only one representative per cluster was used for building the phylogenetic tree (Tables S7 and S8). The number of sequences per cluster is indicated in parentheses. Sequences identified in this study are labeled with black circles (if all sequences of the cluster originate from this work) or gray circles (if the cluster also contains sequences from UniProt). Clusters without circles mean that all sequences are from UniProt.

Structurally, NODs strongly resemble qNORs (66), and to some extent the NorB subunit. In our data, we were able to detect many NorBC with metagenomics, but only 28 on the protein level (Fig. 4, colored triangle), and none of these expressed ones were predicted to be NOD (having the H-to-N mutation). This number is generally low compared to the other enzymes involved in denitrification, presumably because NorBC (and NODs) are membrane proteins and therefore difficult to detect with proteomics. While the NODs were originally described in *Methylomirabilota*, no bacteria of this phylum were detected in our samples. This is not surprising, given the fact that our cultures were enriched on lignocellulose as a carbon source and *Methylomirabilota* are methanotrophs that presumably use the oxygen produced by NOD to oxidize methane to methanol in anoxia. Almost two-thirds of our *nod* genes were found in *Pseudomonadota*, followed by *Bacteroidota* and a few other phyla. Phylogenetic analysis revealed that the NOD sequences clustered into three different clades, to a large degree overlapping with their taxonomic origin (Fig. 5)—the only exception being NODs from *Pseudomonadota,* which were found in two different clades. Generally, the high number of *nod* genes in *Pseudomonadota* was surprising, as other phyla are more commonly associated with NODs (72). The phylogenetic tree structure using our NODs resembles previous phylogenetic analyses using data from sequence repositories; however, while we detect one clade being monophyletic of *Bacteroidota* NODs, this clade is split into two *Bacteroidota* clades in Ruff et al. (72).

None of the species found to have a *nod* gene in the lakes are known methanotrophs, suggesting that if NODs do produce O_2_, it must serve functions other than methane oxidation in these communities. The metabolic purpose of any NOD-derived O_2_ in these lake enrichments remains an open question. Oxidative lignocellulose-degrading enzymes such as LPMOs or lignin-active oxidases represent one speculative possibility; however, the absence of co-expressed AA enzymes in NOD-containing MAGs (except MAG.0909) argues against a direct functional coupling between NOD and oxidative lignocellulose degradation in this data set. Whether any O_2_ produced intracellularly could be transferred to O_2_-consuming enzymes in neighboring cells also remains uncertain.

In *M. oxygeniifera*, the genes of the particulate methane monooxygenase (*pmoA-C*) are located in close proximity to the two putative *nod* genes (Fig. S3, bottom). To elucidate other possible metabolic functions of the oxygen produced by NODs in the lake enrichments, we examined the gene neighborhood of our *nod* genes (Fig. S3). Unfortunately, no clear oxygen-consuming enzyme systems such as the pMMO in *M. oxygeniifera* could be identified in immediate *nod* proximity, leaving this question open for further research. However, the level of genomic similarity within bacterial orders is striking; *Burkholderiales* consistently have two cytochrome C-peroxidases next to *nod*, as well as a methylamine utilization cluster (*mauABED*). In FEN-1191 (*Steroidobacteriales*), *nod* is surrounded by genes encoding iron complex outer-membrane receptor proteins (TC.FEV.OM), possibly involved in the uptake of ferric iron or heme (NOD has two heme groups), as well as K07234, which is, according to KEGG, an uncharacterized protein involved in response to NO. The presence of carnitine CoA-ligase (*caiC*) and a monoterpene epsilon-lactone hydrolase (*mlhB*) might suggest the degradation of terpenoid and aliphatic compounds for these bacteria—it is intriguing to speculate whether O_2_ produced by NOD may be involved in the process. It is also interesting that these loci contain relevant transcriptional regulators. For example, in FEN-1191, we observe *nsrR*, a regulator of genes involved in cellular protection against NO, while in *Polaromonas*, we find *oxyR*, known for the expression of antioxidant genes in response to oxidative stress. Similar regulators (LuxR) have also been detected previously in *M. oxygeniifera* and Gammaproteobacterium HdN1, and in *nod*-containing *Cecembia calidifontis* (NsrR) (78), possibly suggesting a role for NOD in NO detoxification.

### Concluding remarks

This meta-omics study provides a detailed characterization of the strategies employed by microbial communities for lignocellulose transformation under denitrifying conditions. The most dominant lignocellulose degraders—also among the most abundant MAGs throughout all lake enrichments—did not belong to the core MAGs, that is, the MAGs present in all lake enrichments. This observation suggests a degree of lake-specific microbial specialization, likely reflecting the local composition and availability of lignocellulose in the various lake shorelines, consistent with the core-satellite species hypothesis (55). Collectively, these dominant degraders encoded a broad spectrum of CAZymes, targeting multiple fractions of lignocellulose and highlighting their capacity for complex polymer decomposition. We note, however, that substrate-free enrichment controls were not included in the experimental design; while the sub-culturing strategy, slow gas kinetics, visual changes to the spruce, and breadth of expressed CAZymes collectively support lignocellulose-driven metabolism, a contribution from residual organic matter in the inoculum or labile carbon in the added spruce cannot be formally excluded.

Regarding coupled carbon and nitrogen metabolism, DNRA organisms appeared generally better equipped to degrade complex polysaccharides than canonical denitrifiers. Nevertheless, in these complex microbial communities, denitrifiers appear to be sustained by utilizing the simpler carbon compounds produced by other community members, consistent with metabolic interdependence between functional guilds—a hypothesis supported by the meta-omics data but not directly demonstrated in the current study.

Interestingly, a substantial fraction of MAGs did not encode denitrification pathways; however, it persisted throughout the 48-week anoxic enrichment. These organisms may be non-denitrifying anaerobes respiring alternative electron acceptors such as sulfate or they may derive energy via fermentation (54). Consistent with this, our metagenomic data set includes MAGs with complete dissimilatory sulfate reduction pathways (*dsrAB* and *aprAB*), assigned to genera such as *Sulfuricella* and *Polaromonas*, as well as MAGs encoding thiosulfate oxidation coupled to denitrification, suggesting that sulfur cycling contributes to energy conservation alongside carbon and nitrogen transformations in these enrichments.

Strikingly, several MAGs in the eutrophic lake enrichments harbored *nod* genes, although only two belonged to the same phylogenetic clade where NODs were originally described (methanotrophic bacteria). This data set therefore provides a large repertoire of novel *nod*-containing MAGs, potentially with alternative functional roles in utilizing the internally produced O_2_—if this truly is the function of NODs.

Overall, this study advances our understanding of metabolic interactions in complex microbial communities degrading lignocellulose under denitrifying conditions originating from lake sediments. These insights may inform strategies to improve water quality, protect aquatic ecosystems, and mitigate greenhouse gas emissions, while also shedding new light on the ecological and biochemical roles of the enigmatic NO dismutases.

## Data Availability

Raw shotgun metagenomic data have been deposited to the European Nucleotide Archive with the accession number PRJEB102493. The mass spectrometry proteomics data have been deposited to the ProteomeXchange Consortium via the PRIDE (80) partner repository with the data set identifier PXD069160. All annotated prokaryote MAGs and the HMM used to extract NOD sequences are available publicly at 10.6084/m9.figshare.30590648.

## SUPPLEMENTAL MATERIAL

Supplemental material msystems.00577-26-s0001.docx Tables S1-S3 and S5; Fig S1-S3.

Supplemental tables msystems.00577-26-s0002.xlsx Tables S4 and S6-S9.

ASM does not own the copyrights to Supplemental Material that may be linked to, or accessed through, an article. The authors have granted ASM a non-exclusive, world-wide license to publish the Supplemental Material files. Please contact the corresponding author directly for reuse.
