# Supplementary material for: Microbial consortia mediating lignocellulose turnover and denitrification in eutrophic lake sediment enrichments
Source: mSystems. 2026 Jun 29;11(7):e00577-26. doi: 10.1128/msystems.00577-26 (PMC13386854; doi:10.1128/msystems.00577-26)
Supplement: Supplemental material — Tables S1-S3 and S5; Fig S1-S3. [file msystems.00577-26-s0001.docx]

**Microbial consortia mediating lignocellulose turnover and denitrification in eutrophic lake sediment enrichments**

Valerie C. Schiml^1^, Kaja Stalder^1^, Anikó Várnai^1^, Linda L. Bergaust^1^, Lars R. Bakken^1^, Magnus Ø. Arntzen^1*^

^1^Faculty of Chemistry, Biotechnology and Food Science, Norwegian University of Life Sciences (NMBU), P.O. Box 5003, N-1432, Ås, Norway.

**SUPPLEMENTARY TABLES**

**Table S1** – Locations of eutrophic lakes used in this study

**Table S2** – Carbohydrate-active enzymes detected at protein level that may take part in anaerobic lignocellulose degradation

**Table S3** – Carbohydrate-active enzymes detected at protein level that may take part in interaction between microbial species

**Table S4** – Metagenome-assembled genomes (MAGs) - [Excel file]

**Table S5** – Unique species detected in eutrophic- or control lake enrichments.

**Table S6 –** Identified proteins from metaproteomics **-** [Excel file]

**Table S7 –** Protein sequences used to build the phylogenetic tree (Figure 5) **-** [Excel file]

**Table S8 –** Protein sequences within the different subclusters in Figure 5 **-** [Excel file]

**Table S9 –** Assembly statistics **-** [Excel file]

**SUPPLEMENTARY FIGURES**

**Figure S1**: Nitrogen-gas production

**Figure S2**: Measures of quality for metagenomics and metaprotomics

**Figure S3**: Gene neighborhoods of *nod* genes

**SUPPLEMENTARY TABLES**

**Table S1. Locations of eutrophic lakes used in this study.** The table shows the freshwater lakes used in this study. The lakes were selected based on the amount of surrounding agricultural farmland and having a eutrophic status according to measurements performed by the Norwegian government’s implementation of the EU Water Framework Directive. The two control lakes were forest lakes located >100 altitude meters above any farmland. All lakes were sampled in August 2020. Both lake water and sediment were collected.

| **Lake no.** | **Local name** | **Lake status** | **Initial pH** | **Temperature (°C)** | **Coordinates** | **Water type** | **Sampling depth (cm)** |
| --- | --- | --- | --- | --- | --- | --- | --- |
| 1 | Østensjøvann, fiskeplassen | Eutrophic | 6.0 | 20 | 59.686287 N, 10.826634 E | Still | 15 |
| 2 | Bølstadbekken | Eutrophic | 6.3 | 20 | 59.688507 N, 10.818011 E | Running | 80 |
| 3 | Skibekken | Eutrophic | 6.4 | 17 | 59.700051 N, 10.836735 E | Running fast | 10 |
| 4 | Langetjernet | Control | 4.4 | 19 | 59.706933 N, 10.954054 E | Still | 20 |
| 5 | Orremåsan | Control | 4.3 | 18 | 59.705930 N, 10.970153 E | Still | 10 |
| 6 | Årungen Syd | Eutrophic | 6.4 | 22 | 59.676107 N, 10.746479 E | Still | 40 |
| 7 | Årungen Nord | Eutrophic | 6.3 | 23 | 59.692714 N, 10.734506 E | Running slow | 150 |
| 8 | Sæbyvannet, bekken | Eutrophic | 6.3 | 20 | 59.429506 N, 10.969347 E | Running slow | 20 |
| 9 | Flesjøvannet | Eutrophic | 5.5 | 19 | 59.442073 N, 10.953349 E | Still | 50 |
| 10 | Vansjø, Huggernes | Eutrophic | 5.8 | 21 | 59.403354 N, 10.773210 E | Still | 20 |
| 11 | Bjørkelangen | Eutrophic | 5.8 | 18 | 59.841823 N, 11.496050 E | Still | 20 |
| 12 | Østensjøvann Nord | Eutrophic | 6.4 | 18 | 59.689066 N, 10.821406 E | Still | 40 |

**Table S2:** **Carbohydrate-active enzymes detected at protein level that may take part in anaerobic lignocellulose degradation.** The table organizes the detected CAZymes based on their predicted activity and target substrate and provides the identified CAZy modules in the domain structure and the MAGs expressing such proteins. In addition, proteins of unknown function carrying CBM47, CBM44, CBM26, or CBM57 (i.e., without identification of catalytic CAZyme modules) were detected; these proteins are not listed in the table. Abbreviations: AA, auxiliary activity; CBM, carbohydrate-binding module; GH, glycoside hydrolase; GT, glycoside transferase; PL, polysaccharide lyase; CE, carbohydrate esterase.

| Substrate | Predicted enzyme activity | CAZy annotation | MAGs |
| --- | --- | --- | --- |
| Cellulose | Cellobiohydrolase, reducing end-acting | GH48 | MAG.0346, MAG.0092, MAG.0083 |
|  |  | GH48;CBM2 | MAG.0618, MAG.0330 |
|  |  | GH48;CBM3 | CMAG.21, CMAG.01 |
|  | Cellobiohydrolase, non-reducing end-acting | CBM64;GH6 | MAG.0093, MAG.0126 |
|  | Endo-β-1,4-glucanase | GH5^1^ | MAG.0158, MAG.0146 |
|  |  | GH5_1;CBM3 | CMAG.21, CMAG.01 |
|  |  | GH5_2 | MAG.1060, MAG.0857, MAG.0714, MAG.0642 |
|  |  | GH5_4^1^ | CMAG.21, CMAG.01 |
|  |  | GH5_5 | MAG.0714 |
|  |  | GH5_25 | MAG.0642 |
|  |  | GH5_39 | MAG.0978 |
|  |  | GH5_55^1^ | MAG.1060 |
|  |  | GH6 | MAG.1163, MAG.1005, MAG.0926, MAG.0813, MAG.0714, MAG.0705, MAG.0642, MAG.0552, MAG.0346, MAG.0330, MAG.0310, MAG.0146, MAG.0116 |
|  |  | GH6;CBM2 | MAG.0759 |
|  |  | GH8^1^ | MAG.1060, MAG.1005, MAG.0997, MAG.0857, MAG.0714, MAG.0705, MAG.0642, MAG.0604, MAG.0552, MAG.0523, MAG.0492, MAG.0251, MAG.0146, MAG.0092, MAG.0083, CMAG.04 |
|  |  | GH12^1^ | MAG.1060, MAG.1005, MAG.0926, MAG.0899, MAG.0857, MAG.0714, MAG.0705, MAG.0642, MAG.0604, MAG.0552, MAG.0523, MAG.0492, MAG.0251, MAG.0158, MAG.0092, MAG.0083 |
|  |  | GH44^1^ | MAG.0714, MAG.0705, MAG.0492, MAG.0458, MAG.0092 |
|  |  | GH51_3 | MAG.0552, MAG.0492 |
|  |  | CBM8;CBM8;GH51_3 | MAG.1184, MAG.0216 |
|  |  | GH148 | MAG.1045, MAG.0083 |
|  | Endo-β-1,4-glucanase, processive | GH9 | MAG.0857, MAG.0714, MAG.0346, MAG.0092 |
|  |  | GH9;CBM3;CBM3 | CMAG.21, CMAG.01 |
|  |  | CBM4;GH9 | MAG.0997, MAG.0978, MAG.0926, MAG.0714, MAG.0705, MAG.0642, MAG.0604, MAG.0492, MAG.0251, MAG.0092 |
|  |  | CBM30;GH9 | MAG.0092 |
|  | Exo-β-1,4-glucanase / cellodextrinase^2^ | GH5_53 | MAG.0735, MAG.0126 |
|  | Multifunctional endo-β-1,4-glucanase, exo-β-1,4-glucanase and cellobiohydrolase | GH12;GH5_53;CBM3;GH6 | MAG.0093 |
|  | Cellodextrin phosphorylase | GH94 | MAG.1099, MAG.0879, MAG.0801, MAG.0714, MAG.0535, MAG.0524, MAG.0464, MAG.0363, MAG.0362, MAG.0306, MAG.0216, MAG.0011, CMAG.01 |
|  | Bifunctional cellodextrin phosphorylase and cyclic β-1,2-glucan synthase | GT84;GH94 | CMAG.07 |
|  | β-Glucosidase, non-reducing end-acting^2^ | GH3 | MAG.1135, MAG.1060, MAG.0978, MAG.0926, MAG.0899, MAG.0857, MAG.0642, MAG.0552, MAG.0523, MAG.0492, MAG.0346, MAG.0343, MAG.0251, MAG.0159, MAG.0146, MAG.0092, MAG.0083, MAG.0074, MAG.0002, CMAG.13, CMAG.11, CMAG.08, CMAG.05 |
|  | Lytic polysaccharide monooxygenase | AA10;CBM2 | MAG.0618 |
|  | FAD-dependent gluco-oligosaccharide oxidase | AA7 | MAG.0286, MAG.0353, MAG.0888 |
|  | Glucose oxidase/Glucose dehydrogenase | AA3_2^3^ |  |
|  | Pyrroloquinoline quinone-dependent (glucose/sorbosone) dehydrogenase | AA12 | MAG.1002, MAG.0989, MAG.0492, MAG.0146, MAG.0092, MAG.0083, MAG.0079 |
| Xyloglucan | (Xyloglucan-specific) endo-β-1,4-glucanase | GH5_4^1^ |  |
|  |  | GH12^1^ |  |
|  |  | GH44^1^ |  |
|  | α-Xylosidase | GH31^4^ | MAG.1329 |
|  |  | GH31_3 | MAG.0450 |
|  |  | GH31_4 | MAG.1146, MAG.0524 |
|  | β-Galactosidase, terminal non-reducing | GH2^5^ | MAG.0830, MAG.0328, MAG.0007 |
|  |  | GH35^5^ | MAG.0524 |
|  | α-L-Fucosidase | GH29^5^ | MAG.0821 |
|  |  | GH29;CBM32 | MAG.0108 |
|  |  | GH95^5^ | MAG.0327, MAG.0279, MAG.0163 |
|  |  | GH95;CBM35^5^ | CMAG.04 |
|  | Acetylesterase | CE20 | MAG.0393 |
| Mixed-linkage β‑glucan | Endo-β-1,3-glucanase | CBM56;GH64 | CMAG.21, CMAG.01 |
|  |  | CBM38;GH81;CBM6 | MAG.0125 |
|  |  | GH128 | MAG.0450, MAG.0092 |
|  | Endo-β-1,4-glucanase^1^ |  |  |
|  | Exo-β-1,4-glucanase / cellodextrinase^2^ |  |  |
|  | β-Glucosidase, non-reducing end-acting^2^ |  |  |
| Laminarin (brown algae) | Glucan 1,3-β-glucosidase | GH55_1 | CMAG.04 |
| (Galacto) ‑ (gluco)mannan | Endo-β-1,4-mannanase | GH5_8 | MAG.1060, MAG.0714, MAG.0092, MAG.0083 |
|  |  | GH5_8;CBM13 | MAG.0757, MAG.0216 |
|  |  | GH5_10 | MAG.0705, MAG.0642, MAG.0492 |
|  |  | GH5_17 | MAG.0146 |
|  |  | GH5_55^1^ | MAG.1060 |
|  |  | GH26 | MAG.1192, MAG.0857, MAG.0714, MAG.0646, MAG.0642, MAG.0586, MAG.0158, MAG.0146, MAG.0083 |
|  |  | GH26;CBM23;CBM23 | MAG.0618 |
|  |  | CBM35;GH26;CBM46;CBM3 | CMAG.01 |
|  |  | CBM35;GH26;CBM46;CBM46;CBM3 | CMAG.21 |
|  |  | CBM35;CBM35;CBM35;GH26 | MAG.0871, MAG.0757 |
|  |  | CBM54;GH26;CBM27;CBM23 | CMAG.18 |
|  |  | CBM54;GH26;CBM23;CBM59 | CMAG.01 |
|  |  | CBM54;GH26;CBM27;CBM23;CBM59 | CMAG.04 |
|  |  | GH134 | MAG.0642, MAG.0092, MAG.0083 |
|  | β-Mannosidase, terminal non-reducing | GH2 | CMAG.11 |
|  | β-Glucosidase, terminal non-reducing^2^ |  |  |
|  | β-1,4-Mannooligosaccharide phosphorylase | GH130_2 | MAG.1192, MAG.0879, MAG.0646, MAG.0641, MAG.0497, MAG.0384, MAG.0339, MAG.0216, CMAG.21, CMAG.01 |
|  | β-1,4-Mannosylglucose phosphorylase | GH130_1 | MAG.0646, MAG.0641, MAG.0497, MAG.0480, MAG.0363, MAG.0231, MAG.0216, MAG.0159, CMAG.01 |
|  | β-Mannoside phosphorylase | GH130_11 | MAG.0706 |
|  | α-Galactosidase | GH27^6^ | MAG.1192, MAG.1155, MAG.1066, MAG.0646, MAG.0497, MAG.0493, MAG.0216 |
|  |  | GH36^6^ | MAG.0476, CMAG.01 |
| Xylan | Endo-β-1,4-xylanase | GH5_21;GH5_35 | CMAG.21 |
|  |  | GH10 | MAG.1150, MAG.1149, MAG.1045, MAG.0714, MAG.0642, MAG.0324, MAG.0146 |
|  |  | GH10;CBM2 | MAG.0759 |
|  |  | GH10;CBM6;CBM22;CBM22 | MAG.0492 |
|  |  | GH10;CBM6;CBM22;CBM22;CBM22 | MAG.0552, MAG.0346, MAG.0092 |
|  |  | GH10;CBM22;CBM22;CBM22 | MAG.0926 |
|  |  | CBM22;CBM22;GH10;CBM9 | CMAG.21 |
|  |  | CBM22;CBM22;GH10;CBM9;CBM22;GH10;CBM9 | CMAG.01 |
|  |  | CBM85;GH10 | MAG.0899, MAG.0642, MAG.0552, MAG.0251, MAG.0092 |
|  |  | GH11 | MAG.0997, MAG.0857, MAG.0705, MAG.0642, MAG.0552, MAG.0492, MAG.0346, MAG.0251, MAG.0083, CMAG.21, CMAG.01 |
|  |  | GH30^7^ | MAG.1060, MAG.1045, MAG.1005, MAG.0978, MAG.0926, MAG.0705, MAG.0642, MAG.0604, MAG.0577, MAG.0552, MAG.0492, MAG.0251, MAG.0092, MAG.0083 |
|  | (Glucuronoxylan) endo-β-1,4-xylanase | GH30_8 | MAG.0714, MAG.0146 |
|  |  | GH30_8;CBM35 | CMAG.01 |
|  | β-1,4-Xylosidase | GH30^7^ |  |
|  |  | GH30_2 | MAG.1060, MAG.1005, MAG.0978, MAG.0926, MAG.0899, MAG.0857, MAG.0811, MAG.0705, MAG.0642, MAG.0604, MAG.0552, MAG.0523, MAG.0492, MAG.0251, MAG.0158, MAG.0092, MAG.0083 |
|  |  | GH52 | MAG.0092 |
|  | Acetylxylan esterase | CE2 | MAG.1060, MAG.0926, MAG.0642, MAG.0523, MAG.0492, MAG.0346, MAG.0251, MAG.0216, MAG.0092, MAG.0083 |
|  |  | CE3 | MAG.0857, MAG.0492, MAG.0216, MAG.0092 |
|  |  | CE4^8^ | MAG.0705, MAG.0552, CMAG.11 |
|  |  | CE4;CBM36^8^ | CMAG.21, CMAG.01 |
|  |  | CE6 | MAG.0092 |
|  |  | CE7 | CMAG.01 |
|  | Acetylxylan esterase / Feruloyl esterase | CE1 | MAG.1060, MAG.0714, MAG.0642, MAG.0492, MAG.0114, MAG.0092, MAG.0083, CMAG.27, CMAG.17, CMAG.01 |
|  | Bifunctional acetylxylan esterase and polysaccharide synthase | CE4;GT2^8^ | MAG.0705 |
|  | α-L-Arabinofuranosidase, non-reducing end-active | GH43_16;CBM6 | MAG.0492, MAG.0092, MAG.0083 |
|  |  | GH51_1^9^ | MAG.1329, MAG.1091, MAG.0450, CMAG.13, CMAG.01 |
|  |  | GH54^9^ | MAG.1005, MAG.0926, MAG.0642, MAG.0492, MAG.0158 |
|  |  | GH62^9^ | MAG.0714, MAG.0642, MAG.0604, MAG.0552, MAG.0251, MAG.0146, MAG.0092, MAG.0083 |
| (Glucurono)-xylan | α-Glucuronidase | GH4^10^ |  |
|  |  | GH67 | MAG.0642, MAG.0092 |
|  |  | GH115 | MAG.1045 |
| Lignin–carbohydrate complexes | (4-*O*-Methyl-)glucuronate–lignin esterase | CE15 | MAG.0864, MAG.0714, MAG.0705, MAG.0642, MAG.0586, MAG.0552, MAG.0523, MAG.0492, MAG.0083 |
| Pectin | Pectate lyase | PL1;CBM77 | CMAG.21 |
|  |  | PL1_6 | MAG.1329 |
|  |  | PL3_1 | MAG.0216, CMAG.01 |
|  |  | PL9 | MAG.0857, MAG.0492, MAG.0083 |
|  | Endo-polygalacturonase | GH28 | MAG.1184, MAG.0492, MAG.0363 |
|  | Rhamnogalacturonan lyase | PL11 | MAG.0857 |
|  |  | PL9 |  |
|  | Bifunctional rhamnogalacturonan endolyase and pectin acetylesterase | PL11;CE12;CE12 | CMAG.21 |
|  | Exo-poly-α-digalacturonosidase, non-reducing end-active | GH28 |  |
|  | Pectate disaccharide-lyase (exopolygalacturonate lyase); reducing end-active | PL22 |  |
|  | Oligogalacturonate lyase | PL22 | MAG.0495 |
|  | D-4,5-Unsaturated α-galacturonidase (Unsaturated rhamnogalacturonyl hydrolase) | GH105 | MAG.0523, MAG.0492, MAG.0260, MAG.0083 |
|  | Pectin methylesterase | CE8 | MAG.1329, MAG.0714, MAG.0346, MAG.0146, CMAG.08 |
|  | Pectin acetylesterase | CE12 | MAG.0714 |
|  | Endo-β-1,2-apiosidase | GH140 | MAG.0871, MAG.0646 |
|  | Endo-β-1,4-galactanase | CBM61;GH53 | MAG.0586 |
|  | β-Galactosidase, terminal non-reducing | GH35^5^ |  |
|  | β-L-Arabinofuranosidase | GH27^6^ |  |
|  |  | GH36^6^ |  |
|  | α-L-Arabinofuranosidase, non-reducing end-active | GH51_1^9^ |  |
|  |  | GH54^9^ |  |
|  |  | GH62^9^ |  |
| Lignin | Laccase | AA1 | MAG.1233, MAG.0197, MAG.0089, MAG.0013, CMAG.07 |
|  | GMC oxidoreductase | AA3 | MAG.0642, MAG.0592, MAG.0492, MAG.0353, CMAG.22, CMAG.11 |
|  | Aryl alcohol oxidase / aryl alcohol dehydrogenase | AA3_2^3^ | MAG.0977, MAG.0736, MAG.0734, MAG.0502, MAG.0495, MAG.0333, MAG.0090 |
|  | Vanillyl-alcohol oxidase | AA4 | MAG.0909, MAG.0807, MAG.0495, MAG.0465, MAG.0408, MAG.0230, MAG.0175 |
|  | NADPH:*p*-benzoquinone oxidoreductase | AA6 | MAG.0492, MAG.0416, MAG.0404, MAG.0172, MAG.0092, MAG.0083, MAG.0067, MAG.0039, CMAG.22, CMAG.13, CMAG.08 |
| Starch | α-Amylase | GH13_1 | CMAG.21, CMAG.18, CMAG.01 |
|  |  | GH13_6 | MAG.1329 |
|  |  | GH13_19 | CMAG.11 |
|  | α-Amylase / neopullulanase | GH13_46 | MAG.1329 |
|  | Isoamylase (Debranching enzyme) | GH13_11 | CMAG.11 |
|  | Pullulanase (Limit dextrinase) | GH13_13;GH13_13 | MAG.0397 |
|  |  | CBM41;GH13_13 | MAG.1329, CMAG.11 |
|  | Bifunctional α-Amylase and pullulanase | CBM41;CBM41;GH13_12;GH13_41 | CMAG.01 |
|  | Glucoamylase (terminal non-reducing ends of starch polysaccharides) | GH15 | MAG.0129 |
|  | α-1,4-Glucan phosphorylase | GT35 | MAG.0714, MAG.0492, MAG.0201, MAG.0104, MAG.0092, CMAG.27, CMAG.11, CMAG.08, CMAG.01 |
|  | α-Glucosidase (terminal non-reducing ends of malto-oligosaccharides) | GH13_38 | MAG.0171 |
|  |  | GH31^4^ |  |
|  |  | GH97 | MAG.0475 |
|  | Maltose-6′-phosphate glucosidase; α,α-trehalose-6-phosphate glucosidase | GH4^10^ | MAG.0476, CMAG.29 |
|  | 4-α-Glucanotransferase (dextrin glycosyltransferase) | GH77 | MAG.0434, MAG.0116, MAG.0073, CMAG.08 |
|  | Starch synthase (maltosyl transferring) | GH13_3 | MAG.0561, MAG.0092, MAG.0083 |
|  | Glycogen synthase | GT5 | MAG.0714, MAG.0216, CMAG.11 |
|  | α-1,4-Glucan branching enzyme [amylo-(1,4→1,6)-transglycosylase] | GH13_9 | MAG.1155, MAG.0801, MAG.0540 |
|  |  | CBM48;GH13_9 | CMAG.11 |
|  | Cyclomaltodextrin glucanotransferase | GH13_2;CBM20 | CMAG.24 |
|  | Malto-oligosyltrehalose synthase | GH13_26 | MAG.0106 |
|  | Malto-oligosyltrehalose trehalohydrolase | GH13_10 | MAG.1294, MAG.1164, MAG.1138, MAG.0492, MAG.0475, MAG.0450, MAG.0185 |
|  | α,α-Trehalase | GH37 | CMAG.08 |
|  | Trehalose-6-P synthase | GT20 | MAG.0604, MAG.0056 |

1) Some enzyme classes with endo-β-1,4-glucanase activity may exhibit activity towards substrates other than cellulose, including xyloglucans, xylans, mannans, and/or chitosan. Corresponding CAZyme domains and MAGs are listed at cellulose.

2) Some exo-β-1,4-glucanases and β-glucosidases also hydrolyse β-(1→4)-linked D-glucosyl residues from the nonreducing end of oligosaccharides from mixed-linkage β-glucan and glucomannan. Corresponding CAZyme domains and MAGs are listed at cellulose.

3) Some AA3_2 enzymes listed under aryl alcohol oxidases or dehydrogenases may be glucose oxidases or dehydrogenases.

4) GH31 enzymes hydrolyse α-xylosides in xyloglucan, α-glucosides in starch or α-*N*-acetylgalactosaminides in O-glycans. Corresponding MAGs are listed at xyloglucan.

5) α-L-fucosidases and β-galactosidases hydrolyse α-L-fucosyl and β-D-galactosyl substitutions, which are common in xyloglucan, in O-glycans of proteins and, the latter one also, in pectic galactan. Corresponding MAGs are listed at xyloglucan.

6) Some GH27 and GH36 enzymes listed under α-galactosidases at galacto(gluco)mannan may be β-L-arabinosidases hydrolysing pectic arabinan and arabinogalactan. Corresponding MAGs are listed at galacto(gluco)mannan.

7) HMMer annotation does not specify subfamily; some GH30 members listed under endo-β-1,4-xylanases may be β-xylosidases or endo-β-1,6-glucanases.

8) CE4 enzymes deacetylate xylosyl residues in acetylxylan or *N*-acetylglucosamine residues in peptidoglycan, chitin or chito-oligosaccharides. Corresponding MAGs are listed at xylan.

9) α-L-arabinofuranosidases hydrolyse the terminal arabinosyl substitutions in arabinoxylan, arabinan and arabinogalactan. Corresponding MAGs are listed at xylan.

10) GH4 enzymes include maltose-6′-phosphate glucosidases, α-glucuronidases, α-galactosidases, 6-phospho-β-glucosidases, or α-glucosidases. Only the first two (most prevalent) activities are indicated in this table. Corresponding MAGs are listed at starch.

**Table S3: Carbohydrate-active enzymes detected at protein level that may take part in interaction between microbial species.** The table organizes the detected CAZymes based on their predicted activity and target substrate and provides the identified CAZy modules in the domain structure and the MAGs expressing such proteins. In addition, proteins of unknown function carrying CBM47, CBM44, CBM26, or CBM57 (i.e., without identification of catalytic CAZyme modules) were detected; these proteins are not listed in the table. Abbreviations: AA, auxiliary activity; CBM, carbohydrate-binding module; GH, glycoside hydrolase; GT, glycoside transferase; PL, polysaccharide lyase; CE, carbohydrate esterase.

| Substrate | Predicted enzyme activity | CAZy annotation | MAGs |
| --- | --- | --- | --- |
| Bacterial cell wall (peptidoglycan) | Lysozyme | GH19_1 | MAG.0159 |
|  |  | GH23 | CMAG.26, CMAG.11 |
|  |  | GH25 | MAG.0492 |
|  | Peptidoglycan lytic transglycosylase / peptidoglycan lyase | GH102 | MAG.0052 |
|  |  | GH103 | MAG.0145 |
|  | Peptidoglycan β-*N*-acetylmuramidase, terminal non-reducing ends | GH171 | MAG.0282 |
|  | Peptidoglycan glycosyltransferase | GT51 | MAG.1250, MAG.0708, MAG.0312, MAG.0019, CMAG.13, CMAG.11, CMAG.07 |
|  | Peptidoglycan *N*-acetylglucosamine deacetylase | CE4^8^ |  |
|  |  | CE4;CBM36^8^ |  |
|  | Bifunctional polysaccharide synthase and peptidoglycan *N*-acetylglucosamine deacetylase | CE4;GT2^8^ |  |
|  | *N*-Acetylglucosamine-6-phosphate deacetylase | CE9 | MAG.0652, CMAG.11 |
| Bacterial cell wall (D-arabinan) | Exo-α-1,5-D-arabinofuranosidase | GH172 | MAG.1105 |
| Fungal cell wall (chitin, chitosan) | Chitinase | GH23 | CMAG.26, CMAG.11 |
|  | Chitosanase | GH8^1^ |  |
|  | Chitin and chitooligosaccharide deacetylase | CE4^8^ |  |
|  | Chitooligosaccharide deacetylase | CE14 | MAG.0122, MAG.0110 |
| Mucopolysaccharides / Glycosaminoglycans | Hyaluronate lyase / Chondroitin lyase | PL8 | CMAG.01 |
|  |  | PL33_1 | MAG.0037 |
| Glycoproteins, *O*-glycans | Exo-α-sialidase | GH177 | MAG.0483, MAG.0480, CMAG.11 |
|  | α-L-Fucosidase | GH29^5^ |  |
|  |  | GH95^5^ |  |
|  |  | GH95;CBM35^5^ |  |
|  | β-Galactosidase, terminal non-reducing | GH2^5^ |  |
|  | α-*N*-Acetylgalactosaminidase | GH31^4^ |  |
|  |  | GH109 | MAG.1045, MAG.0384, MAG.0252, MAG.0208, MAG.0108, CMAG.11 |
|  | α-Mannosyltransferase | GT105 | MAG.0038 |
| Glycoproteins, N-glycans | α-1,4-Mannosidase, non-reducing end-acting | GH92 | MAG.1329 |
|  | β-*N*-Acetylhexosaminidase, terminal non-reducing ends | GH179 | MAG.0927, MAG.0705, MAG.0646 |
| Other | Glycosyltransferases, various substrates | GT1 | MAG.0251, MAG.0092 |
|  |  | GT2 | MAG.0705, MAG.0502, MAG.0492, MAG.0157, MAG.0146, CMAG.11 |
|  |  | GT4 | MAG.1106, MAG.0791, MAG.0381, MAG.0346, MAG.0258, MAG.0215, MAG.0118, MAG.0012, CMAG.12 |
|  | UDP-ManNAc: β-*N*-acetyl-mannosaminyltransferase | GT26 | MAG.0146 |
|  | 4-Amino-4-deoxy-L-arabinotransferase | GT83 | MAG.0310 |
|  | UDP-3-*O*-acyl-*N*-acetylglucosamine deacetylase | CE11 | MAG.0604 |

1) Some enzyme classes with endo-β-1,4-glucanase activity may exhibit activity towards substrates other than cellulose, including xyloglucans, xylans, mannans and/or chitosan. Corresponding CAZyme domains and MAGs are listed at cellulose.

2) Some exo-β-1,4-glucanases and β-glucosidases also hydrolyse β-1,4-linked D-glucosyl residues from the nonreducing end of oligosaccharides from mixed-linkage β-glucan and glucomannan. Corresponding CAZyme domains and MAGs are listed at cellulose.

3) Some AA3_2 enzymes listed under aryl alcohol oxidases or dehydrogenases may be glucose oxidases or dehydrogenases.

4) GH31 enzymes hydrolyse α-xylosides in xyloglucan, α-glucosides in starch or α-*N*-acetylgalactosaminides in *O*-glycans. Corresponding MAGs are listed at xyloglucan.

5) α-L-fucosidases and β-galactosidases hydrolyse α-L-fucosyl and β-D-galactosyl substitutions, which are common in xyloglucan, in *O*-glycans of proteins and, the latter one also, in pectic galactan. Corresponding MAGs are listed at xyloglucan.

6) Some GH27 and GH36 enzymes listed under α-galactosidases at galacto(gluco)mannan may be β-L-arabinosidases hydrolysing pectic arabinan and arabinogalactan. Corresponding MAGs are listed at galacto(gluco)mannan.

7) HMMer annotation does not specify subfamily; some GH30 members listed under endo-β-1,4-xylanases may be β-xylosidases or endo-β-1,6-glucanases.

8) CE4 enzymes deacetylate xylosyl residues in acetylxylan or *N*-acetylglucosamine residues in peptidoglycan, chitin or chito-oligosaccharides. Corresponding MAGs are listed at xylan.

9) α-L-arabinofuranosidases hydrolyse the terminal arabinosyl substitutions in arabinoxylan, arabinan and arabinogalactan. Corresponding MAGs are listed at xylan.

10) GH4 enzymes include Maltose-6′-phosphate glucosidases, α-glucuronidases, α-galactosidases, 6-phospho-β-glucosidases, α-glucosidases or. Only the first two (most prevalent) activities are indicated in this table. Corresponding MAGs are listed at starch.

**Table S4: Metagenome-assembled genomes (MAGs).**

This table is available as a separate Excel-file.

**Table S5: Unique species detected in eutrophic- or control lake enrichments.** The table lists the species and MAGs detected with metagenomics uniquely present in either the eutrophic lake enrichments or in the control lake enrichments.

| **Unique Species**  **Eutrophic lake enrichments** | **MAGs** |
| --- | --- |
| *Polaromonas sp023259235* | MAG.0191, MAG.0376, MAG.0519, MAG.0571, MAG.0893, MAG.1068, MAG.1276, MAG.1214 |
| *Afipia sp024707075* | MAG.0356, MAG.0832, MAG.0681, MAG.0945, MAG.1265, MAG.1119 |
| *Giesbergeria hankyongi* | MAG.0172, MAG.0472, MAG.0501, MAG.0875, MAG.1193, MAG.1158 |
| *Giesbergeria sp017985015* | MAG.0207, MAG.0283, MAG.0776, MAG.0947, MAG.1001, MAG.1041 |
| *Kaistella soli* | MAG.0185, MAG.0358, MAG.0375, MAG.1267, MAG.1162, MAG.1326 |
| *Phaeospirillum sp900184795* | MAG.0448, MAG.0465, MAG.0866, MAG.1287, MAG.1210, MAG.1213 |
| *Acidovorax delafieldii_B* | MAG.0204, MAG.0329, MAG.0799, MAG.1180, MAG.1108 |
| *Cellulomonas gelida* | MAG.0618, MAG.0658, MAG.0759, MAG.1048 |
| *Dokdonella sp001899855* | MAG.0566, MAG.0907, MAG.0719, MAG.0951 |
| *Thiobacillus thioparus* | MAG.0213, MAG.0298, MAG.0385, MAG.0410 |
| *Cellulomonas soli* | MAG.0396, MAG.0449, MAG.0840 |
| *Geothrix sp019449415* | MAG.0428, MAG.0504, MAG.1250 |
| *Thermomonas beijingensis* | MAG.0365, MAG.0739, MAG.1285 |
| Fen-183 sp016716715 | MAG.0282, MAG.0882 |
| *Geothrix sp020622065* | MAG.1126, MAG.1147 |
| *Giesbergeria sp003096555* | MAG.0026, MAG.0906 |
| *Thermomonas sp024498955* | MAG.0648, MAG.0669 |
| *Undibacterium aquatile* | MAG.0458, MAG.1143 |
| UTCHB3 sp020161395 | MAG.0073, MAG.0652 |
| *Acidovorax radicis_A* | MAG.0878 |
| *Afipia sp000178995* | MAG.0445 |
| *Afipia sp017305455* | MAG.0323 |
| *Afipia sp017308495* | MAG.0395 |
| *Azospirillum melinis* | MAG.0325 |
| *Bosea robiniae* | MAG.1128 |
| CAMDGX01 sp020848935 | MAG.0066 |
| *Castellaniella sp019104865* | MAG.1178 |
| *Cellulomonas sp001898175* | MAG.0122 |
| *Chitinophaga sancti* | MAG.1281 |
| *Desulfobacillus sp018262875* | MAG.0901 |
| *Devosia_A sp017307495* | MAG.0473 |
| *Didemnitutus sp023432225* | MAG.0746 |
| *Dokdonella sp016707485* | MAG.0948 |
| *Dokdonella_A sp019634845* | MAG.0269 |
| *Ferruginibacter sp001898465* | MAG.0587 |
| *Ferruginibacter sp002400445* | MAG.0063 |
| *Flavobacterium inviolabile* | MAG.0312 |
| *Hanamia sp018268075* | MAG.1115 |
| JADKHK01 sp016708465 | MAG.0710 |
| JADLGL01 sp020849325 | MAG.0076 |
| JAEYUG01 sp023432805 | MAG.0253 |
| JAFKJW01 sp023953845 | MAG.1026 |
| JAFLBB01 sp020852175 | MAG.0094 |
| *Mesorhizobium qingshengii* | MAG.0280 |
| OLB17 sp016720705 | MAG.1312 |
| OLB8 sp018057925 | MAG.0795 |
| QKMZ01 sp020854255 | MAG.0673 |
| QY30 sp020622085 | MAG.0048 |
| *Rhodoferax_C sp016705575* | MAG.1025 |
| SCUS01 sp004297725 | MAG.0222 |
| SCUS01 sp016704445 | MAG.0915 |
| *Sediminibacterium sp015999745* | MAG.0511 |
| *Sediminibacterium sp016786865* | MAG.0031 |
| *Sulfuricella sp000971475* | MAG.0127 |
| *Thermomonas sp014678725* | MAG.0911 |
| UBA11398 sp003506065 | MAG.0077 |
| UBA5069 sp002415895 | MAG.0246 |
| UTCHB3 sp016707285 | MAG.0306 |
| VBCG01 sp016704895 | MAG.0614 |
| VBCG01 sp020852555 | MAG.0061 |
| **Unique Species**  **Control lake enrichments** | **CMAGs** |
| *Lacrimispora sp002478865* | CMAG.24 |
| *Methylocella sp003162995* | CMAG.31 |
| *Paenibacillus silagei* | CMAG.21 |
| *Rouxiella badensis* | CMAG.26 |
| *Serratia_A fonticola* | CMAG.11 |

**Table S6: Identified proteins from metaproteomics.**

This table is available as a separate Excel-file.

**Table S7: Protein sequences used to build the phylogenetic tree (Figure 5).**

This table is available as a separate Excel-file.

**Table S8: Protein sequences within the different subclusters in Figure 5.**

This table is available as a separate Excel-file.

**Table S9: Assembly statistics**

This table is available as a separate Excel-file.

**SUPPLEMENTARY FIGURES**


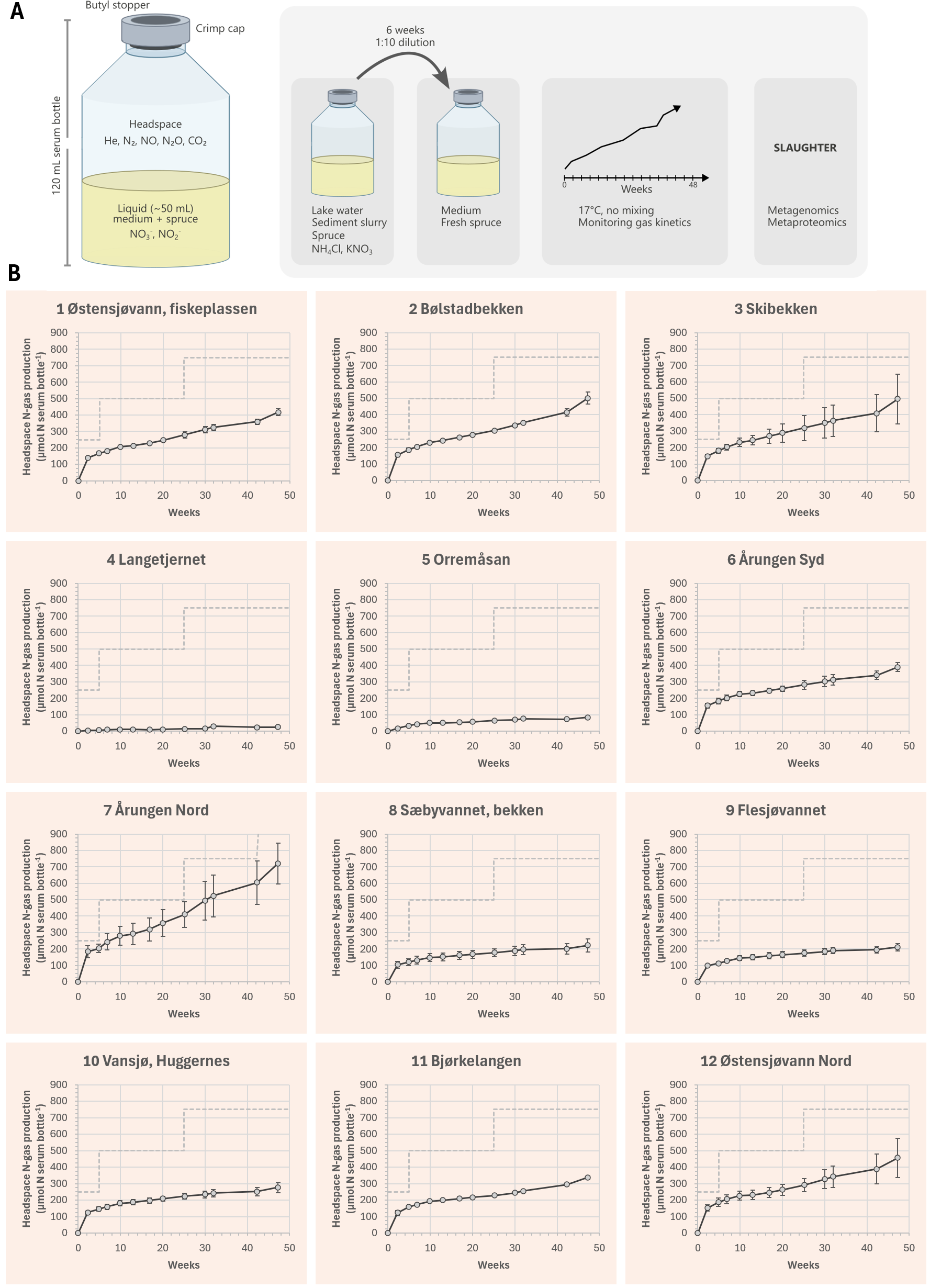


**Figure S1: Experimental design and nitrogen-gas production. A)** An overview of the anaerobic enrichment cultures and experimental design for subculturing, monitoring and slaughter. All samples were as biological triplicates. Metagenomics and metaproteomics was performed on same samples. **B)** Nitrogen-gas production (sum of NO, N_2_O, and N_2_) in enrichment cultures with spruce incubated in closed He-washed serum bottles under denitrifying conditions with 5 mM KNO_3_ for 48 weeks. N-gas production is averaged over three measurements; error-bars represent one standard deviation (n=3). NO and N_2_O accumulated transiently and accounted for a small fraction of the N-gas produced. The dotted lines indicate available N in the bottles, which was increased by adding 5 mM KNO_3_ (250 µmol serum bottle^-1^) twice during the 48-week incubation (three times for lake 7 due to the higher turnover).

In addition to the N-gas production shown, oxyanions (NO_3_⁻ and NO_2_⁻) were measured in the liquid at two timepoints, and the total oxyanion reduction was calculated as the difference between added NO_3_⁻ and residual oxyanions at the end of the enrichment. CO_2_ production was monitored throughout. For the N mass balance: The measured production of gaseous N (NO + N_2_O + N_2_) accounted for 87–116% of the calculated oxyanion reduction, confirming that denitrification was the dominant anaerobic respiratory pathway – DNRA would have reduced this recovery by retaining N as NH_4_⁺.

C and electron balance: The stoichiometry of carbohydrate oxidation yields 4 moles of electrons per mole of carbon oxidized to CO_2_, regardless of whether O_2_ or nitrogen oxides serve as the terminal electron acceptor (one mole of glucose via glycolysis and TCA yields 24 moles of electrons, i.e., 4 mol e⁻ per mol C). The total respiratory electron flux to denitrification (***Fe***) was calculated from the measured reduction of NO_3_⁻ to NO_2_⁻, NO, N_2_O and N_2_, accounting for the electron consumption at each step (2 electrons for NO_3_⁻ → NO_2_⁻; 1 electron per N atom for subsequent steps, totaling 5 electrons for NO_3_⁻ → ½N_2_). The CO_2_ production accountable by denitrification was then estimated as CO_2denitrification_ = ***Fe*** × 0.25. CO_2denitrification_ accounted for 87–116% of measured CO_2_ production across enrichments, indicating that denitrification explained essentially all observed CO_2_ production. We therefore conclude that fermentative metabolism and DNRA were both negligible – if either were significant, measured CO_2_ would substantially exceed CO_2denitrification_.


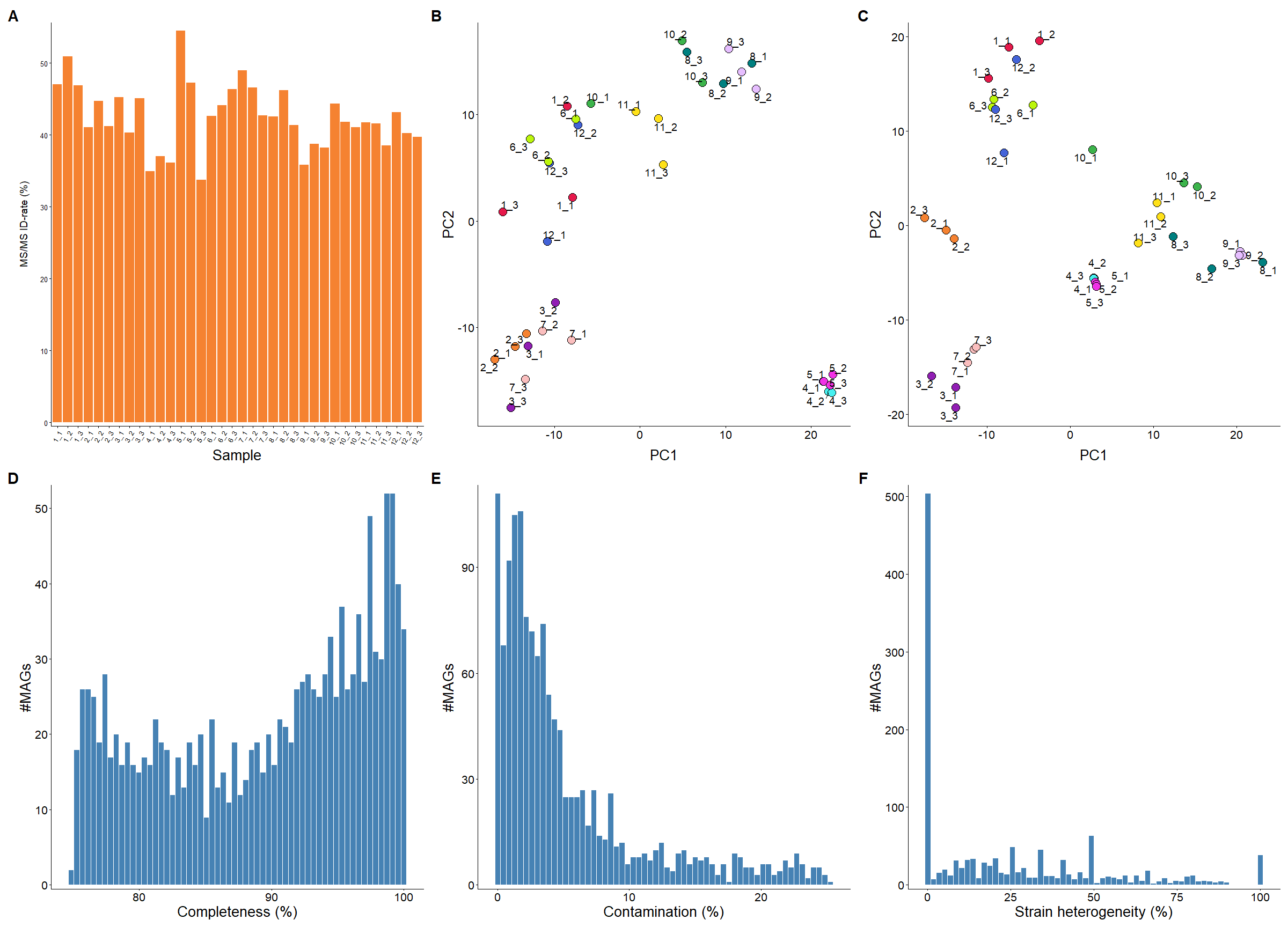


**Figure S2: Measures of quality for metagenomics and metaprotomics.** The figure shows **A)** metaproteomics MS/MS ID-rates, **B)** PCA plot using metagenomics summed LFQ-values per MAG from FragPipe, **C)** PCA plot using metagenomics coverages per MAG from CoverM, **D-F)** measures of MAG quality from CheckM. Note: one single MAG with 68.6% completeness was left out from figure D for visualization purposes, but is available in Table S4.


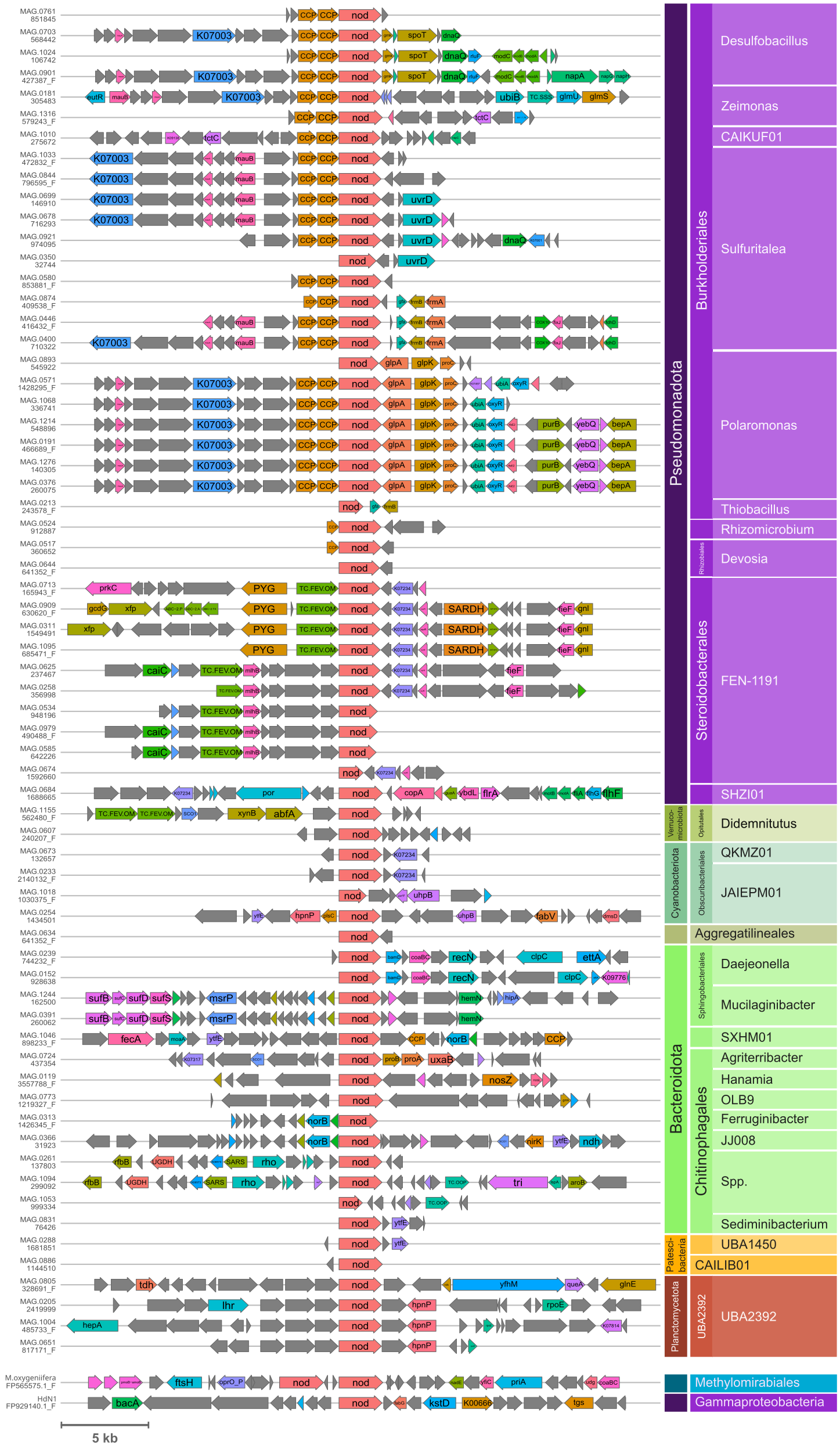


**Figure S3: Gene neighborhood analysis of *nod* genes.** The figure shows the genomic neighborhood of *nod* ± 15 kb in 66 MAGs, aligned together with the *nod*-containing regions of *Methylomirabilis oxygeniifera* FP565575 and Gammaproteobacterium HdN1 FP929140 at the bottom. The *pmoA-C* in *M. oxygeniifera* is shown in purple on the left-hand side. Note that for many of the MAGs, the *nod*-containing contig was relatively short, explaining why there are only a few genes in the proximity of *nod* in these.
